# Supplementary material for: Machine learning identifies key metabolic reactions in bacterial growth on different carbon sources
Source: Mol Syst Biol. 2024 Jan 30;20(3):170–86. doi: 10.1038/s44320-024-00017-w (PMC10912204; doi:10.1038/s44320-024-00017-w)

APPENDIX:

Machine learning identifies key metabolic reactions in bacterial growth on different carbon sources

Hyunjae Woo, Youngshin Kim, Dohyeon Kim and Sung Ho Yoon

Table of Contents

Appendix Text S1 2

Appendix Figure S1 3

Appendix Text S2 4

Appendix Table S1 5

Appendix Text S3 6

Appendix Table S2 11

Appendix Table S3 12

Appendix Table S4 13

Appendix Table S5 14

Appendix Figure S2 15

Appendix Figure S3 30

Appendix Text S1. Evaluation of flux variability analysis and flux sampling as a flux simulation method

In machine learning, values of each feature in each input data need to be specified to prepare and structure the data for training and prediction. In this study, MOMA, an algorithm specifically designed to predict the behavior of a mutant strain (Segrè *et al*, 2002), was used to simulate the metabolic flux distribution of the mutant strain under different carbon conditions. Flux variability analysis (FVA) (Mahadevan & Schilling, 2003) and flux sampling (Herrmann *et al*, 2019) can be alternative methods for flux simulation, offering the advantage of generating non-zero flux values for a larger number of reactions when compared to MOMA. To address this, we applied FVA and flux sampling to generate flux values for glucose condition, by setting the maximum uptake rates of the glucose and oxygen at 10 and 20 mmol/gDCW/h, respectively. Among 2,715 reactions, MOMA generated non-zero flux values for every 435 reactions. FVA yielded non-zero flux values for 565 reactions with at least one non-zero value at the minimum and maximum flux values (Appendix Fig S1A). Within the FVA results, 61 reactions had extremely low minimum flux values (28 ea) (< -990 mmol/gDCW/h), and/or extremely high maximum flux values (44 ea) (> 995 mmol/gDCW/h). Except for these reactions with unrealistic flux values, flux values from MOMA were in good agreement with the minimum flux values from FVA for most reactions (97.8%, 2654 ea) (r = 0.96). Flux sampling generated a nonzero flux distribution for every 1,786 reactions without producing any negative flux values (Appendix Fig S1B). Within the results from flux sampling, 61 reactions had extremely high minimum flux values (26 ea) (> 136 mmol/gDCW/h), and/or extremely high maximum flux values (61 ea) (> 104 mmol/gDCW/h). Even when excluding these reactions with unrealistic flux values, most flux values from MOMA (97.8%, 2654 reactions) showed poor agreement with the minimum (r = 0.22) or average (r = 0.28) flux values obtained from flux sampling.

FVA and flux sampling are designed to capture the potential range of flux values for every metabolic reaction in a metabolic network, rather than finding a single optimal solution. Additionally, as illustrated in Appendix Fig S1, they generated unrealistic flux values for many reactions which could introduce noise or distortions when using this data for machine learning applications. Considering the importance of well-defined and reliable feature values for each data point in machine learning, there's a need to develop new algorithms that enable FVA or flux sampling to generate flux data suitable for machine learning applications.

Appendix Figure S1. Comparison of flux distributions obtained from the flux simulations using MOMA (metabolic simulation of minimization of metabolic adjustment), FVA (flux variance analysis), and flux sampling.

To simulate growth using *in silico* MOPS minimal medium supplemented glucose as the sole carbon source, the maximum uptake rates of the carbon source and oxygen were set at 10 and 20 mmol/gDCW/h, respectively. FVA and flux sampling were performed using the “flux_variability_analysis” function (with default setting) and “OptGPSampler” function (with default setting of 100 iterations), of the COBRApy software (Ebrahim *et al*, 2013), respectively.

A Comparison of flux values from FVA and MOMA simulations. For each of the 565 metabolic reactions with at least one non-zero value at the minimum and maximum flux values in the FVA simulation, the flux range (from minimum to maximum fluxes) in the FVA simulation was compared with the flux value in the MOMA simulation. The y-axis denotes the flux values.

B Comparison of flux values from flux sampling and MOMA simulations. For each of the 1,786 metabolic reactions with as a nonzero flux distribution, the flux distribution in the simulation of flux sampling was compared with the flux value in the MOMA simulation. The left y-axis denotes the flux distributions from the flux sampling simulation as mean $\pm$ standard deviation on a log10 scale. The right y-axis indicates flux values from the MOMA simulation.

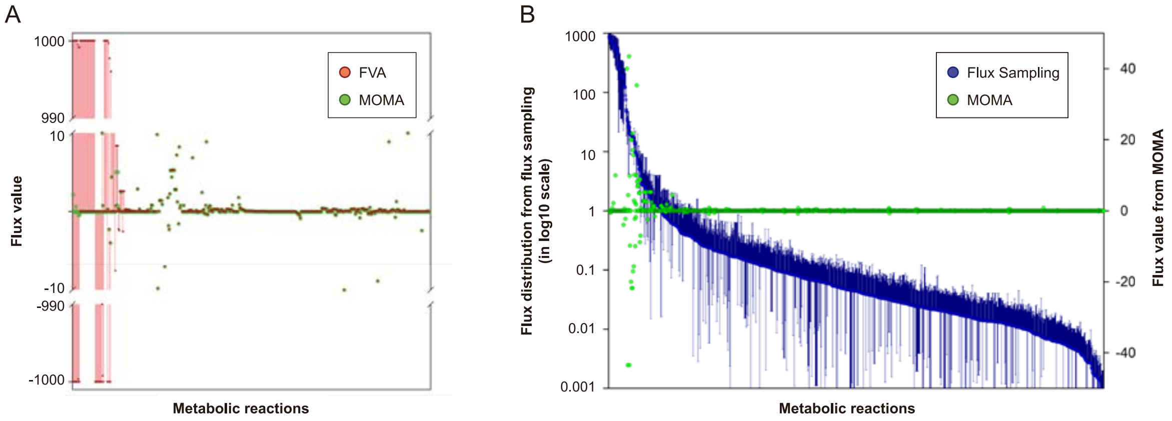

Appendix Text S2. Evaluation of the variance thresholding and random forest as a feature selection method

To increase the performance of machine learning algorithms, feature selection is an important data preprocessing step to reduce the dataset size by eliminating redundant and irrelevant features. The primary goal of feature selection is to identify the optimal subset of features without losing the key features of the data. However, this is an NP-hard (non-deterministic polynomial-time hard) problem (Kohavi & John, 1997), which led to the development of numerous techniques to tackle this issue(Venkatesh & Anuradha, 2019). We tested two commonly used methods of feature selection (Fida *et al*, 2021): the variance thresholding and tree-based random forest on the glucose training dataset.

In our construction of ML models, we removed any features that consistently showed zero values across all mutation simulations. This can be seen as a form of variance thresholding (Fida *et al*, 2021) with threshold set to zero, since all fluxes with constant values had a value of zero, except for only one reaction of the ATP maintenance requirement. For the glucose dataset, the number of selected features decreased from 2715 to 1914. When we implemented variance thresholding with a set threshold of zero, the number of selected features decreased from 2715 to 1913.

Compared to our ML models, variance thresholding with threshold set to one significantly reduced the number of features (from 2715 to 681), and also decreased the accuracy of the MLP model (Appendix Table S1). Applying the random forest algorithm using RandomForestRegressor function with default settings in the sklearn Python package led to only 123 selected features, and also decreased the accuracy of the EN model. Thus, features selected in this study can be considered a subset of features representing salient characteristics of the data, although they might not be the most optimal selection.

Appendix Table S1. Comparison of prediction accuracy of the ML models for the glucose condition using different feature selection methods as a preprocessing step.

	Variance thresholding				Random forest[b]	
	Threshold of 0 (This study)		Threshold of 1[b]			
Feature reduction (ea, before -> after)	2715 -> 1914		2715 -> 681		2715 -> 123	
Model	EN	MLP	EN	MLP	EN	MLP
Beneficial reactions (ea)	291	351	81	188	36	56
Detrimental reactions (ea)	41	24	29	14	11	7
Model accuracy[a]	80.7%	78.7%	80.0%	50.0%	57.1%	77.8%

[a]The accuracy of each model was evaluated by counting the number of critical reactions found among the positive reactions predicted by the model. The critical reactions were obtained from the paper reporting the training dataset for the glucose condition (Tong *et al*, 2020).

[b]Jupyter notebooks for these tasks are available on https://github.com/sybirg/xai_growth/tree/main/Supplementary .

Appendix Text S3: Abbreviations

Terminology

CBM: Constraint-based metabolic modeling
DCW: dry cell weight
DL: Deep learning
EMP: Embden-Meyerhof-Parnas
EN: Elastic net regression
FBA: Flux balance analysis
GEM: Genome-scale metabolic model
ML: Machine learning
MLP: Multilayer perceptron
MOMA: Minimization of metabolic adjustment
PPP: Pentose phosphate pathway
SHAP: SHapley Additive exPlanations
TCA: Tricarboxylic acid

Carbon source

Ac: Acetate
Adn: Adenosine
D-Ala: D-alanine
Fru: Fructose
Fuc: Fucose
Fum: Fumarate
Gal: Galactose
Galr: Galacturonate
Glcn: Gluconate
Gam: Glucosamine
Glc: Glucose
GlcNAc: *N*-Acetyl glucosamine
Glcr: Glucuronate
Gly: Glycerol
Lac: Lactate
L-Ala: L-alanine
Mal: Malate
Malt: Maltose
Mnl: Mannitol
Man: Mannose
NAG: *N*-Acetyl glucosamine
Oaa: Oxaloacetate
Pyr: Pyruvate
Rib: Ribose
Sac: Saccharate
Sbt: Sorbitol

Suc: Succinate
Thy: Thymidine
Tre: Trehalose
Xyl: Xylose
aKG: α-ketoglutarate

Metabolites appeared in Figure 4

2DDG6P: 2-Dehydro-3-deoxy-D-gluconate 6-phosphate
2DDGLCN: 2-Dehydro-3-deoxy-D-gluconate
2DR1P: 2-Deoxy-D-ribose 1-phosphate
2PG: D-Glycerate 2-phosphate
34HPP: 3-(4-Hydroxyphenyl)pyruvate
3IG3P: C'-(3-Indolyl)-glycerol 3-phosphate
3MOB: 3-Methyl-2-oxobutanoate
3PG: 3-Phospho-D-glycerate
3PSEME：5-O-(1-Carboxyvinyl)-3-phosphoshikimate
4ABZ: 4-Aminobenzoate
5MTHF: 5-Methyltetrahydrofolate
6HMHPTPP: 6-hydroxymethyl-dihydropterin pyrophosphate
6HMHPTPP: 6-hydroxymethyl-dihydropterin pyrophosphate
6PGC: 6-Phospho-D-gluconate
6PGL: 6-phospho-D-glucono-1,5-lactone
ACCOA: Acetyl-CoA
AICAR: 5-Amino-1-(5-Phospho-D-ribosyl)imidazole-4-carboxamide
AIR: 5-amino-1-(5-phospho-D-ribosyl)imidazole
AKG: α-ketoglutarate
ALA: Alanine
ARG: Arginine
ASER: O-Acetyl-L-serine
ASN: Asparagine
ASP: Aspartic acid
ASPSA: L-Aspartate 4-semialdehyde
BASP: 4-Phospho-L-aspartate
CHOR: Chorismate
CIT: Citrate
COA: Coenzyme A
CPPPG3: Coproporphyrinogen III
CYS: Cysteine
DCAMP: N6-(1,2-Dicarboxyethyl)-AMP
DHAP: Dihydroxyacetone phosphate
DHF: 7,8-Dihydrofolate
DHNPT: Dihydroneopterin
DHOR_S: (S)-Dihydroorotate
DHPT: Dihydropteroate
DMPP: Dimethylallyl diphosphate
DXYL5P: 1-deoxy-D-xylulose 5-phosphate

E4P: D-Erythrose 4-phosphate
F1P: D-Fructose 1-phosphate
F6P: D-Fructose 6-phosphate
FAD: Flavin adenine dinucleotide oxidized
FAICAR: 5-Formamido-1-(5-phospho-D-ribosyl)imidazole-4-carboxamide
FDP: D-Fructose 1,6-bisphosphate
FGAR: N2-Formyl-N1-(5-phospho-D-ribosyl)glycinamide
FMN: FMN
FUM: Fumarate
G3P: Glyceraldehyde 3-phosphate
G6P: D-Glucose 6-phosphate
GAM: Glucosamine
GAM1P: glucosamine 1-phosphate
GAM1P: D-Glucose 1-phosphate
GAM6P: D-Glucosamine 6-phosphate
GAR: N1-(5-Phospho-D-ribosyl)glycinamide
GlcNAc: *N*-Acetyl glucosamine
GLN: Glutamine
GLU: Glutamate
GLX: Glyoxylate
GLY: Glycine
GLYCLT: Glycolate
GRDP: Geranyl diphosphate
H2MB4P: 1-hydroxy-2-methyl-2-(E)-butenyl 4-diphosphate
Heme b: Protoheme
HIS: Histidine
HSER: L-Homoserine
IASP: Iminoaspartate
ICIT: Isocitrate
IGP: D-erythro-1-(Imidazol-4-yl)glycerol 3-phosphate
ILE: Isoleucine
IMP: IMP
IPDP: Isopentenyl diphosphate
LEU: Leucine
Lipid I: Undecaprenyl-diphospho-N-acetylmuramoyl-L-alanyl-D-glutamyl-meso-2,6-diaminopimeloyl-D-alanyl-D-alanine
Lipid II: Undecaprenyl-diphospho-N-acetylmuramoyl-(N-acetylglucosamine)-L-ala-D-glu-meso-2,6-diaminopimeloyl-D-ala-D-ala
LIPID IVA: KDO-lipid IVA
LYS: Lysine
MET: Methionine
MLTHF: 5,10-Methylenetetrahydrofolate
NAD: Nicotinamide adenine dinucleotide
NAG: *N*-acetyl Glucosamine
NAG6P: N-Acetyl-D-glucosamine 6-phosphate
OAA: Oxaloacetate
ORN: Ornithine

OROT: Orotate
PAP: Adenosine 3',5'-bisphosphate
PAPS: 3'-Phosphoadenylyl sulfate
PEP: Phosphoenolpyruvate
PHE: Phenylalanine
PHP: 3-Phosphohydroxypyruvate
PHPYR: Phenylpyruvate
PPG9: Protoporphyrin
PPHN: Prephenate
PPPG9: Protoporphyrinogen IX
PRLP: 5-[(5-phospho-1-deoxyribulos-1-ylamino)methylideneamino]-1-(5-phosphoribosyl)imidazole-4-carboxamide
PRO: Proline
PRPP: 5-Phospho-alpha-D-ribose 1-diphosphate
PSER: O-Phospho-L-serine
PYR: Pyruvate
R1P: Alpha-D-ribose 1-phosphate
R5P: Alpha-D-ribose 5-phosphate
RL5P: D-Ribulose 5-phosphate
S7P: Sedoheptulose 7-phosphate
SER: Serine
SUCC: Succinate
SUCCOA: Succinyl-CoA
THF: 5,6,7,8-Tetrahydrofolate
THF: 5,6,7,8-Tetrahydrofolate
THR: Threonine
TRP: Tryptophan
TYR: Tyrosine
UDCPP: Undecaprenyl phosphate
UGMDA: UDP-N-acetylmuramoyl-L-alanyl-D-glutamyl-meso-2,6-diaminopimeloyl-D-alanyl-D-alanine
VAL: Valine
X5P: D-Xylulose 5-phosphate

References

Ebrahim A, Lerman JA, Palsson BO, Hyduke DR (2013) COBRApy: COnstraints-Based Reconstruction and Analysis for Python. *BMC Syst Biol* 7: 74

Fida MAFA, Ahmad T, Ntahobari M, 2021. Variance threshold as early screening to Boruta feature selection for intrusion detection system, 2021 13th International Conference on Information & Communication Technology and System (ICTS). pp. 46-50.

Herrmann HA, Dyson BC, Vass L, Johnson GN, Schwartz J-M (2019) Flux sampling is a powerful tool to study metabolism under changing environmental conditions. *NPJ Syst Biol Appl* 5: 32

Kohavi R, John GH (1997) Wrappers for feature subset selection. *Artif Intell* 97: 273-324

Mahadevan R, Schilling CH (2003) The effects of alternate optimal solutions in constraint-based genome-scale metabolic models. *Metab Eng* 5: 264-276

Monk JM, Lloyd CJ, Brunk E, Mih N, Sastry A, King Z, Takeuchi R, Nomura W, Zhang Z, Mori H *et al* (2017) iML1515, a knowledgebase that computes *Escherichia coli* traits. *Nat Biotechnol* 35: 904-908

Otsu N (1979) A threshold selection method from gray-level histograms. *IEEE Trans Syst Man Cybern* 9: 62-66

Segrè D, Vitkup D, Church GM (2002) Analysis of optimality in natural and perturbed metabolic networks. *Proc Natl Acad Sci USA* 99: 15112-15117

Tong M, French S, El Zahed SS, Ong WK, Karp PD, Brown ED (2020) Gene dispensability in *Escherichia coli* grown in thirty different carbon environments. *mBio* 11: e02259-02220

Venkatesh B, Anuradha J (2019) A review of feature selection and its methods. *Cybern Inf Technol* 19: 3-26

Appendix Table S2. Statistics of model predictions of metabolic reactions that positively (+) or negatively (−) affected *E. coli* grown on 30 different carbon sources.

Carbon source	Elastic-net (EN)		Multi-layer perceptron (MLP)		EN ∩ MLP		EN ∪ MLP	
	+	−	+	−	+	−	+	−
Acetate	282	55	351	14	274	3	359	66
Adenosine	278	45	363	5	271	1	370	49
α-Ketoglutarate	297	53	346	5	297	2	346	56
D-Alanine	317	69	349	11	298	1	368	79
Fructose	314	77	351	49	307	8	358	118
Fucose	324	41	349	28	315	1	358	68
Fumarate	322	31	347	5	315	2	354	34
Galactose	358	41	353	9	312	0	399	50
Galacturonate	306	46	352	5	305	0	353	51
Glucosamine	314	51	351	37	301	0	364	88
Glucose	291	41	351	24	283	2	359	63
Glucuronate	311	54	350	11	304	0	357	65
Gluconate	298	47	346	4	298	1	346	50
Glycerol	282	51	350	20	276	7	356	64
L-Alanine	344	61	349	22	312	1	381	82
Lactate	326	56	350	15	314	0	362	71
Malate	318	47	351	19	315	3	354	63
Maltose	316	28	348	18	307	2	357	44
Mannitol	291	36	352	13	281	1	362	48
Mannose	310	52	351	69	301	2	360	119
N-Acetyl glucosamine	307	39	352	29	301	0	358	68
Oxaloacetate	293	53	341	12	281	2	353	63
Pyruvate	326	56	347	5	315	0	358	61
Ribose	311	44	344	8	308	2	347	50
Saccharate	305	51	349	11	300	1	354	61
Sorbitol	305	44	348	40	297	1	356	83
Succinate	331	31	428	1	324	1	435	31
Thymidine	274	44	346	11	271	1	349	54
Trehalose	304	87	369	2	304	1	369	88
Xylose	300	32	362	22	290	2	372	52
Average	308.5	48.8	353.2	17.5	299.2	1.6	362.5	64.6
Standard deviation	18.8	13.0	15.2	15.2	14.7	1.8	17.4	20.4
Total (avg ± std)	357.3 ± 23.0		370.7 ± 19.2		300.8 ± 14.4		427.1 ± 24.3	

Appendix Table S3. ***Escherichia coli*** **strains and plasmids used in this study.**

Strain or plasmid	Description	Source
Strains		
BW25113	Wild-type	Keio Collection
ECK0113 (JW0110)	BW25113 Δ*aceE*::Km^r	Keio Collection
ECK0130 (JW0127)	BW25113 Δ*panD*::Km^r	Keio Collection
ECK0132 (JW0129)	BW25113 Δ*panC*::Km^r	Keio Collection
ECK0133 (JW0130)	BW25113 Δ*panB*::Km^r	Keio Collection
ECK0244 (JW0233)	BW25113 Δ*proA*::Km^r	Keio Collection
ECK0514 (JW0510)	BW25113 Δ*ybcF*::Km^r	Keio Collection
ECK0763 (JW0757)	BW25113 Δ*bioA*::Km^r	Keio Collection
ECK0765 (JW0759)	BW25113 Δ*bioF*::Km^r	Keio Collection
ECK0766 (JW0760)	BW25113 Δ*bioC*::Km^r	Keio Collection
ECK0767 (JW0761)	BW25113 Δ*bioD*::Km^r	Keio Collection
ECK1510 (JW1510)	BW25113 Δ*lsrF*::Km^r	Keio Collection
ECK1511 (JW1511)	BW25113 Δ*lsrG*::Km^r	Keio Collection
ECK1850 (JW1838)	BW25113 Δ*purT*::Km^r	Keio Collection
ECK2019 (JW2006)	BW25113 Δ*hisA*::Km^r	Keio Collection
ECK2306 (JW2309)	BW25113 Δ*purF*::Km^r	Keio Collection
ECK2314 (JW2317)	BW25113 Δ*pdxB*::Km^r	Keio Collection
ECK2431 (JW2429)	BW25113 Δ*hemF*::Km^r	Keio Collection
ECK2496 (JW2485)	BW25113 Δ*purN*::Km^r	Keio Collection
ECK2555 (JW2541)	BW25113 Δ*purL*::Km^r	Keio Collection
ECK2681 (JW2662)	BW25113 Δ*luxS*::Km^r	Keio Collection
ECK3187 (JW3165)	BW25113 Δ*kdsC*::Km^r	Keio Collection
ECK3399 (JW3375)	BW25113 Δ*bioH*::Km^r	Keio Collection
ECK3597 (JW3582)	BW25113 Δ*cysE*::Km^r	Keio Collection
ECK3863 (JW3841)	BW25113 Δ*glnA*::Km^r	Keio Collection
ECK3911 (JW3890)	BW25113 Δ*tpiA*::Km^r	Keio Collection
ECK3931 (JW3910)	BW25113 Δ*metB*::Km^r	Keio Collection
ECK3997 (JW3969)	BW25113 Δ*purD*::Km^r	Keio Collection
ECK4173 (JW4135)	BW25113 Δ*purA*::Km^r	Keio Collection
ECK4380 (JW4351)	BW25113 Δ*serB*::Km^r	Keio Collection
Δ*aceB*Δ*glcB*	BW25113 Δ*glcB*::Km^{r}Δ*aceB*	This study
Plasmids		
pKD3	Chloramphenicol resistance cassette amplification	Addgene
pKD46	λ-red recombinases expression	Lifescience-market

Appendix Table S4. Formulation of *in silico* MOPS minimal media.

The maximum uptake rates of each carbon source and oxygen are set at 10 and 20 mmol/gDCW/h, respectively (Monk *et al*, 2017; Tong *et al.*, 2020). Uptake rates of other components are unbounded.

Metabolite	Reaction ID	Reaction	Lower Bound	Upper Bound
Ammonia	EX_nh4_e	nh4[e] <=>	-1000	1000
Calcium	EX_ca2_e	ca2[e] <=>	-1000	1000
Chloride	EX_cl_e	cl[e] <=>	-1000	1000
Cobalt	EX_cobalt2_e	cobalt2[e] <=>	-1000	1000
Copper	EX_cu2_e	cu2[e] <=>	-1000	1000
ferrous ion	EX_fe2_e	fe2[e] <=>	-1000	1000
Magnesium	EX_mg2_e	mg2[e] <=>	-1000	1000
Manganese	EX_mn2_e	mn2[e] <=>	-1000	1000
Oxygen	EX_o2_e	o2[e] <=>	-20	1000
Phosphate	EX_pi_e	pi[e] <=>	-1000	1000
Potassium	EX_k_e	k[e] <=>	-1000	1000
Sodium	EX_na1_e	na1[e] <=>	-1000	1000
Sulfate	EX_so4_e	so4[e] <=>	-1000	1000
Zinc	EX_zn2_e	zn2[e] <=>	-1000	1000
Carbon source*	Ex_carbon_e	carbon[e] <=>	-10	1000

*Carbon source (exchange reaction): acetate (EX_ac_e), adenosine (EX_adn_e), D-alanine (EX_ala_D_e), fructose (EX_fru_e), fucose (EX_fuc_L_e), fumarate (EX_fum_e), galactose (EX_gal_e), galacturonate (EX_galur_e), gluconate (EX_glcn_e), glucose (EX_glc_D_e), glucosamine (EX_gam_e), glucuronate (EX_glcur_e), glycerol (EX_glyc_e), lactate (EX_lac_D_e), L-alanine (EX_ala_L_e), malate (EX_mal_L_e), maltose (EX_malt_e), mannitol (EX_mnl_e), mannose (EX_man_e), *N*-acetyl glucosamine (EX_acgam_e), oxaloacetate (EX_oaa_e), pyruvate (EX_pyr_e), ribose (EX_rib_D_e), saccharate (EX_glcr_e), sorbitol (EX_sbt_D_e), succinate (EX_succ_e), trehalose (EX_tre_e), thymidine (EX_thymd_e), xylose (EX_xyl_D_e), α-ketoglutarate (EX_akg_e).

Appendix Table S5. Software packages used to build the models and related hyperparameter search value. The selected parameters are underlined. The parameters not mentioned here have default values.

Model	Software	Hyperparameters	Search spaces
**Elastic-net regression**	H2O4GPU	L1-ratio	**0.01**, 0.1, 0.3, 0.5, 0.7, 0.9, 0.99
		cv	300
		Max iteration	1e4
		Tolerance	1e-6
**Multi-layer perceptron**	Keras and Tensorflow	Number of hidden layers	1, 2, 3, **4**
		Number of nodes per layer	5, 10, 25, 50, 100, 200, **1000**, 2000
		Dropout rate	0.3, 0.4, 0.5, **0.6**
		Kernel constraint	None, max_norm(2), max_norm(3), **max_norm(4)**
		Optimizer	SGD, ADAM, **RMSprop**
		Learning rate	0.1, 0.05, 0.01, **0.005**, 0.001, 0.0001
		Activation function	ReLU
		Epoch	40
		Execution per trial	3
		Max trials	10000
		Tuner objective	val_mse
		Random seed	0

Appendix Figure S2. Mapping of the model prediction onto metabolic pathways for each of 30 carbon source conditions.

The solid arrows indicate single metabolic reactions, and dashed arrows indicate multiple sequential reaction steps. The color scheme of each arrow indicates the impact of each metabolic reaction on cell growth: red, beneficial; blue, detrimental; black, not significant.

Acetate

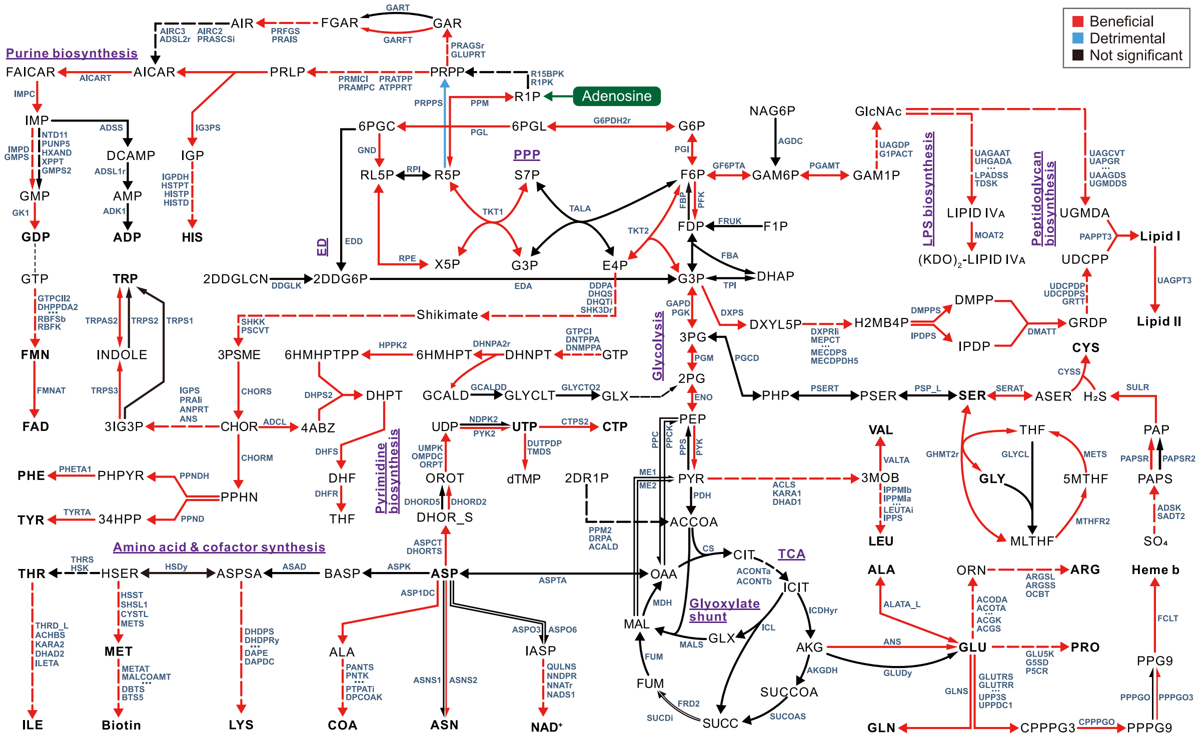

Adenosine

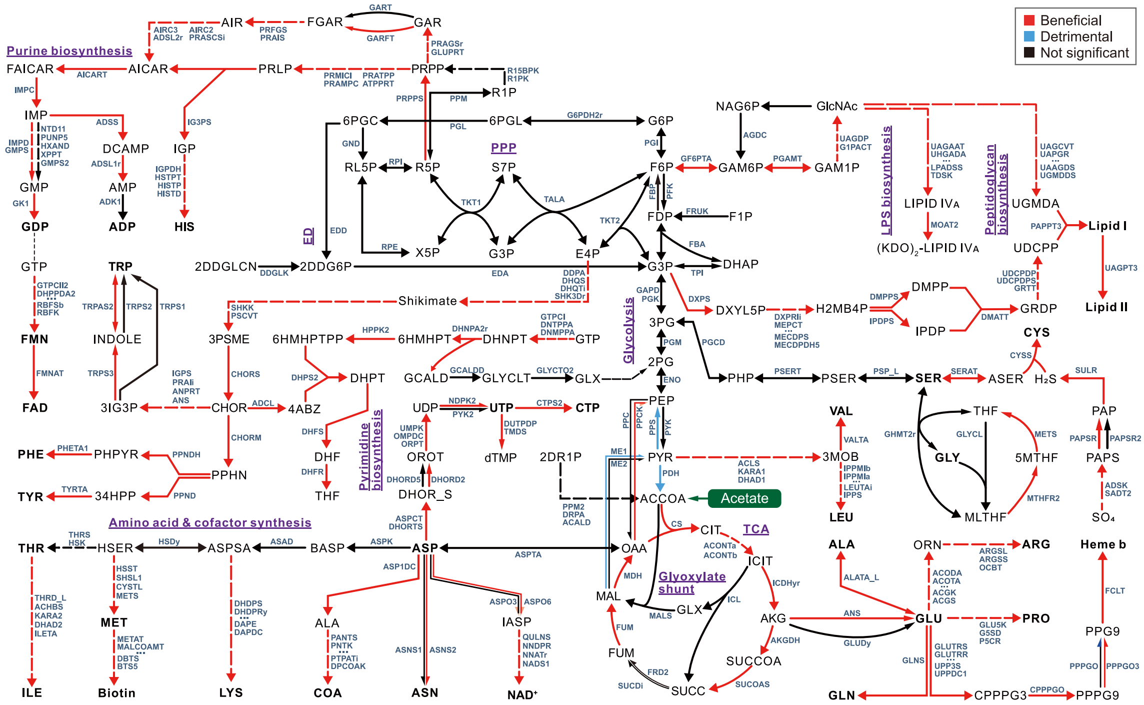

α-ketoglutarate

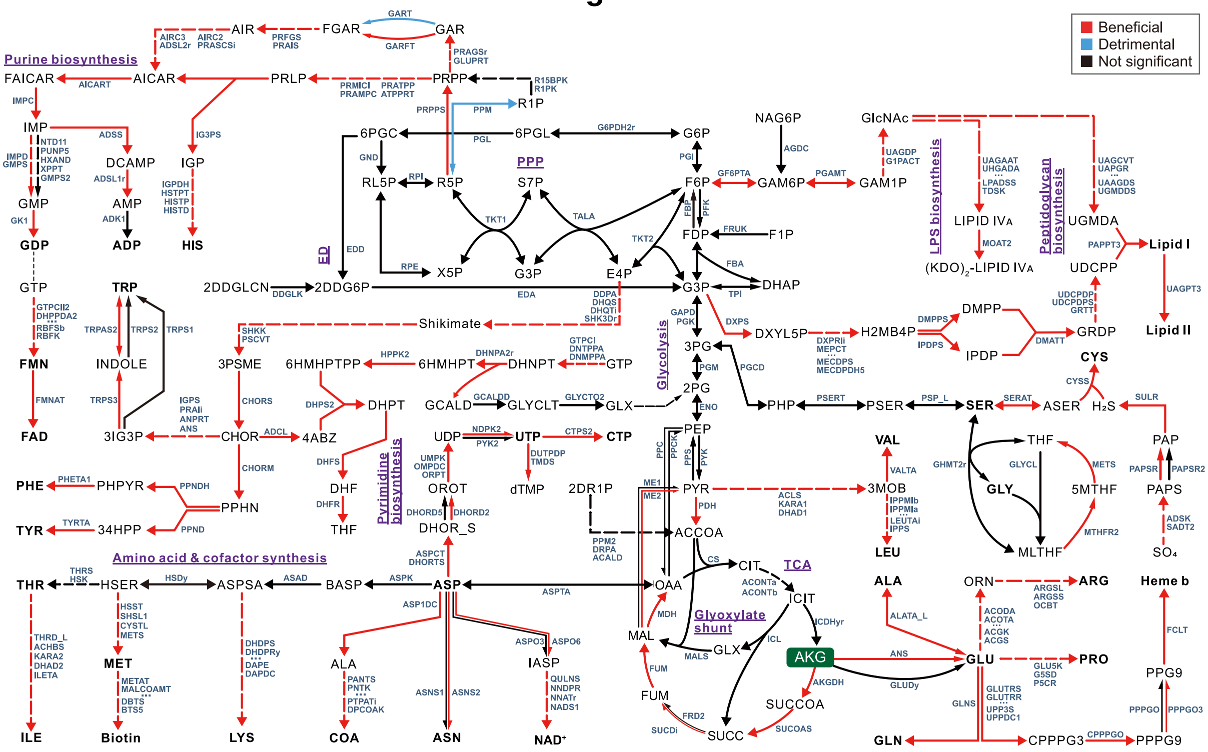

D-Alanine

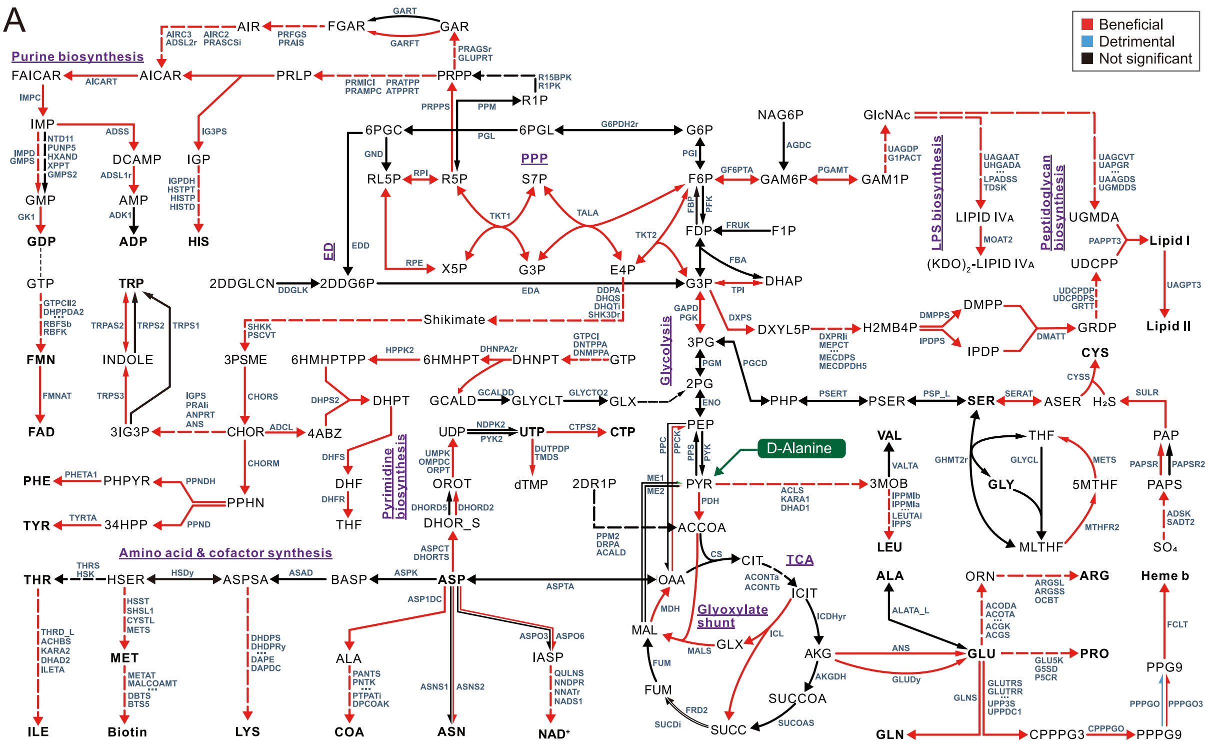

Fructose

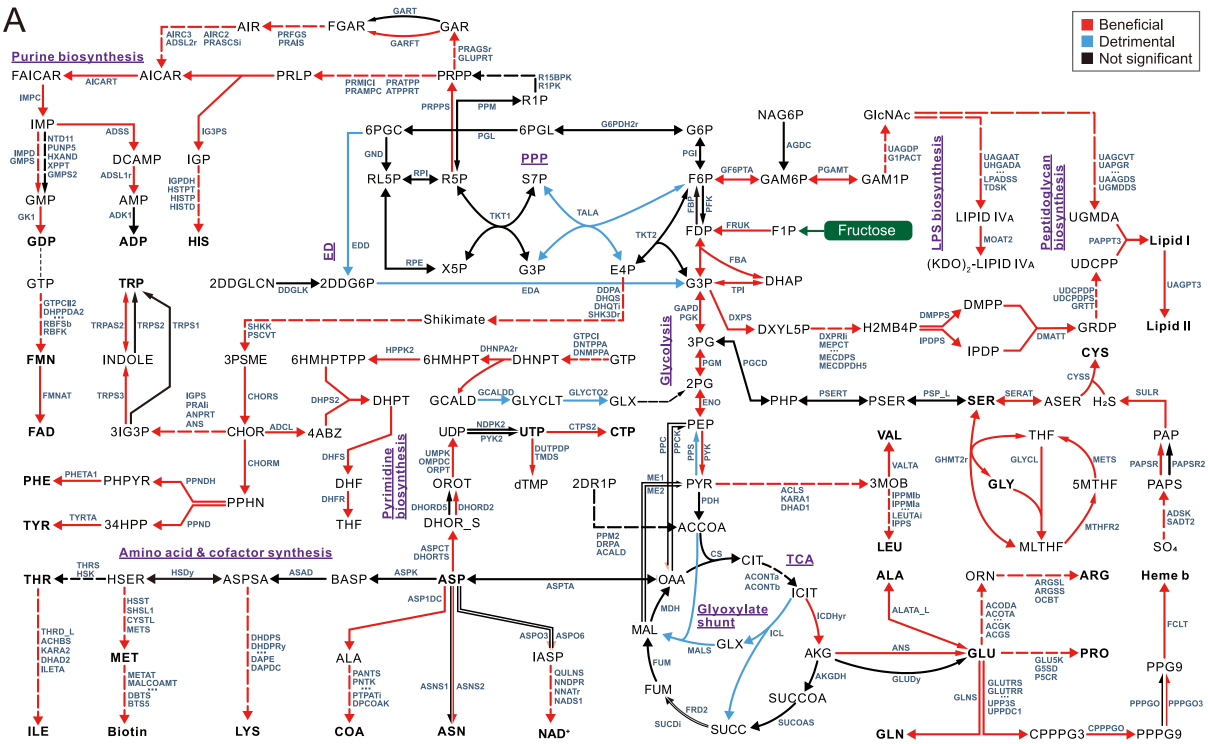

Fucose

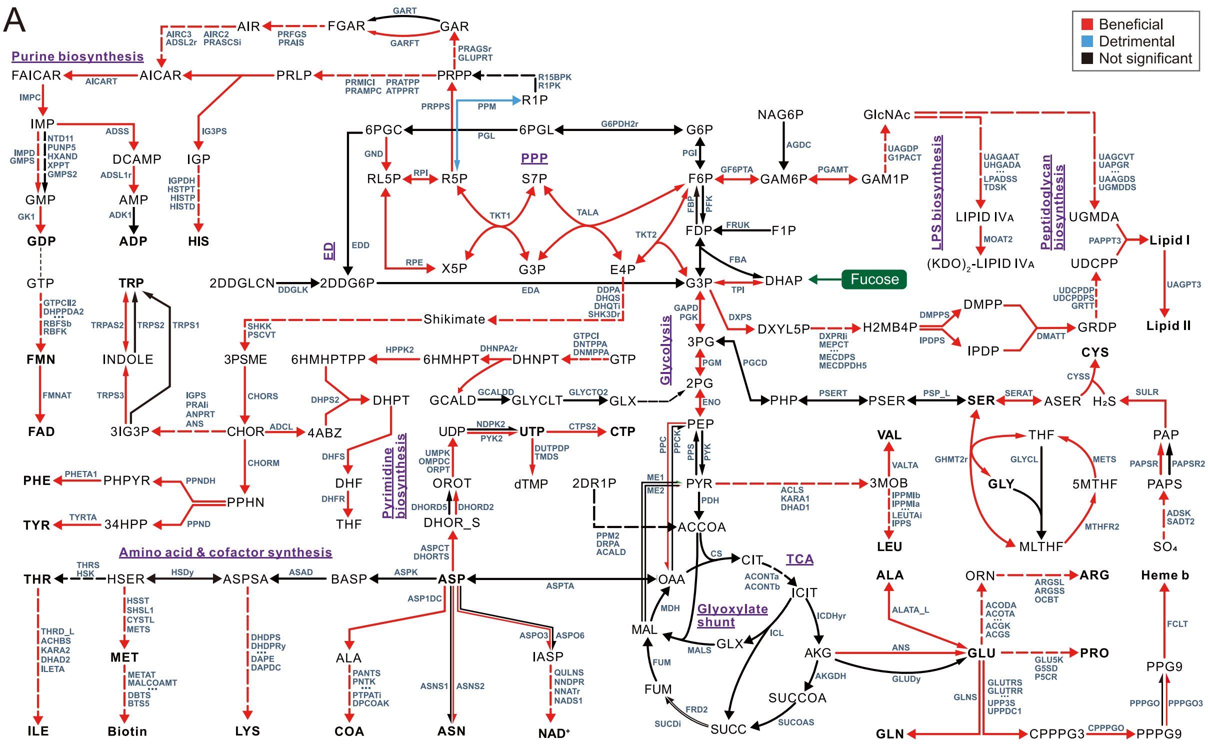

Fumarate

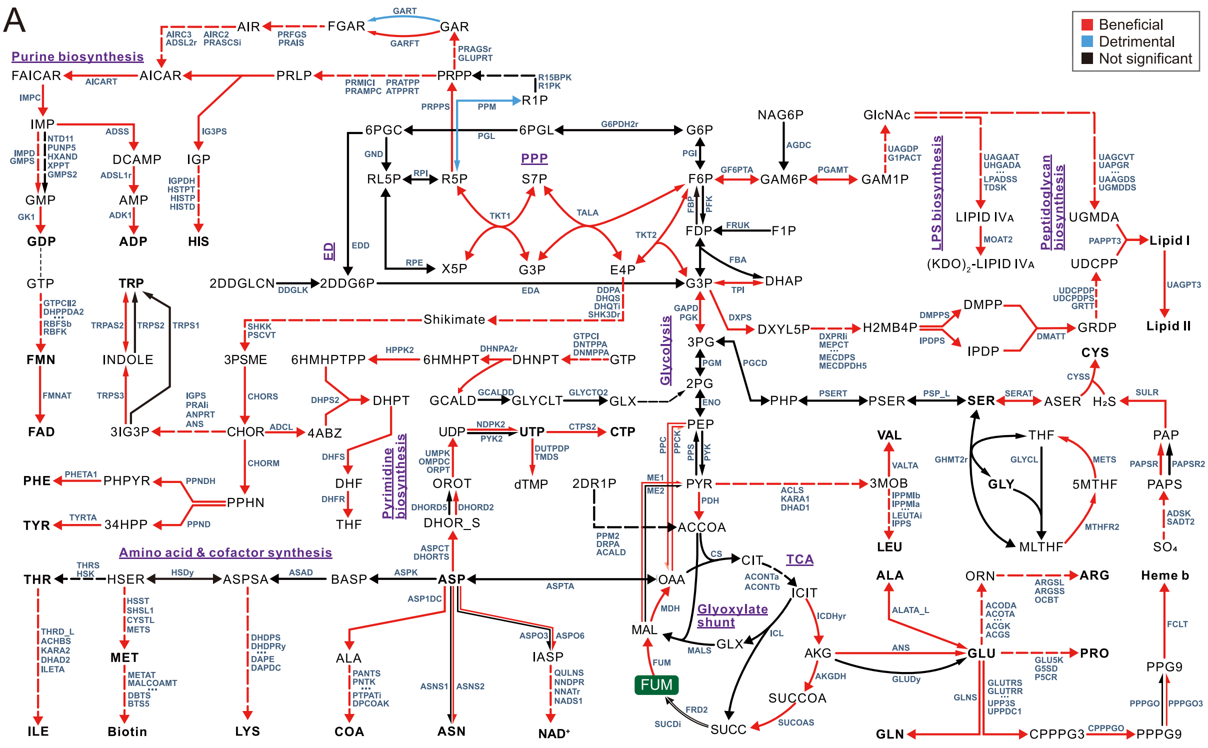

Galactose

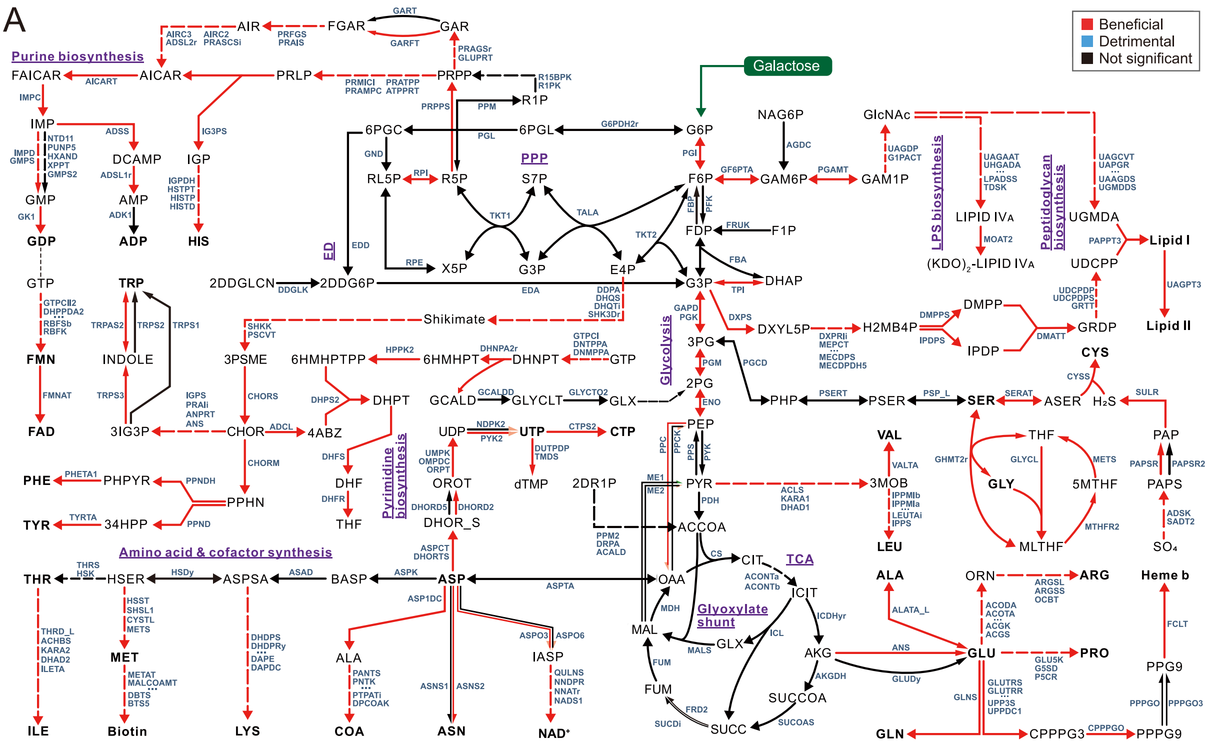

Galacturonate

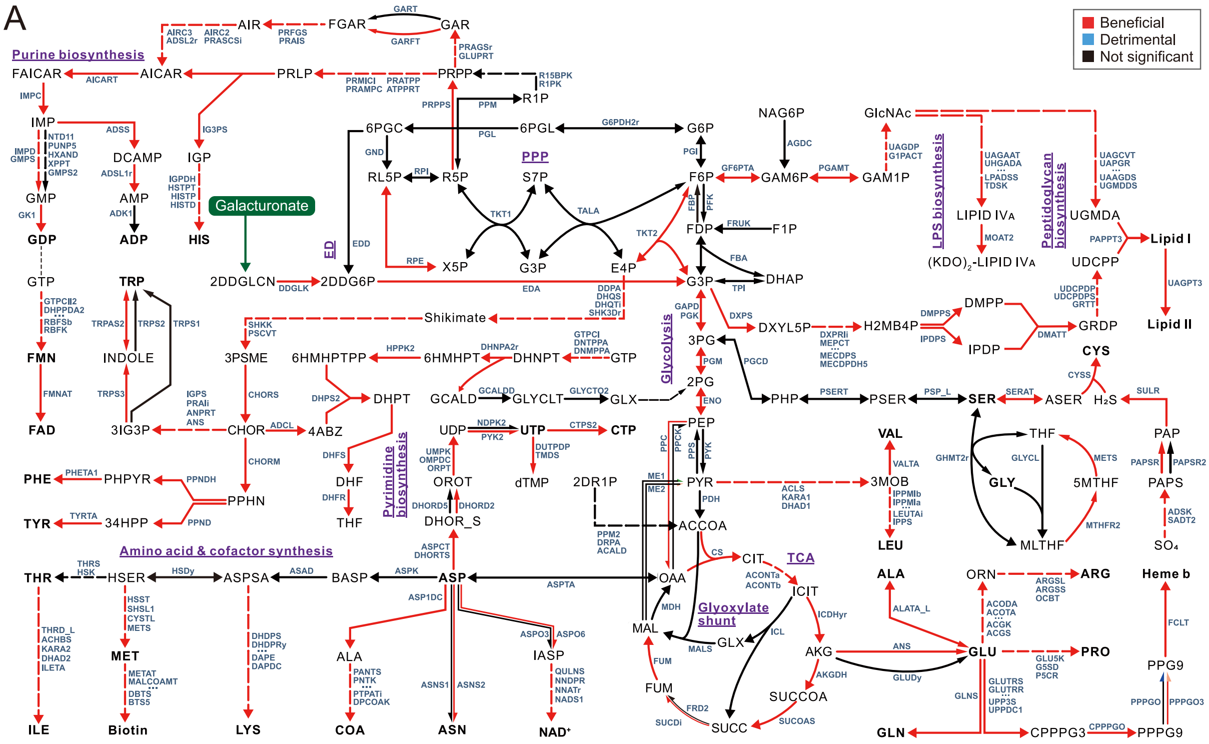

Glucosamine

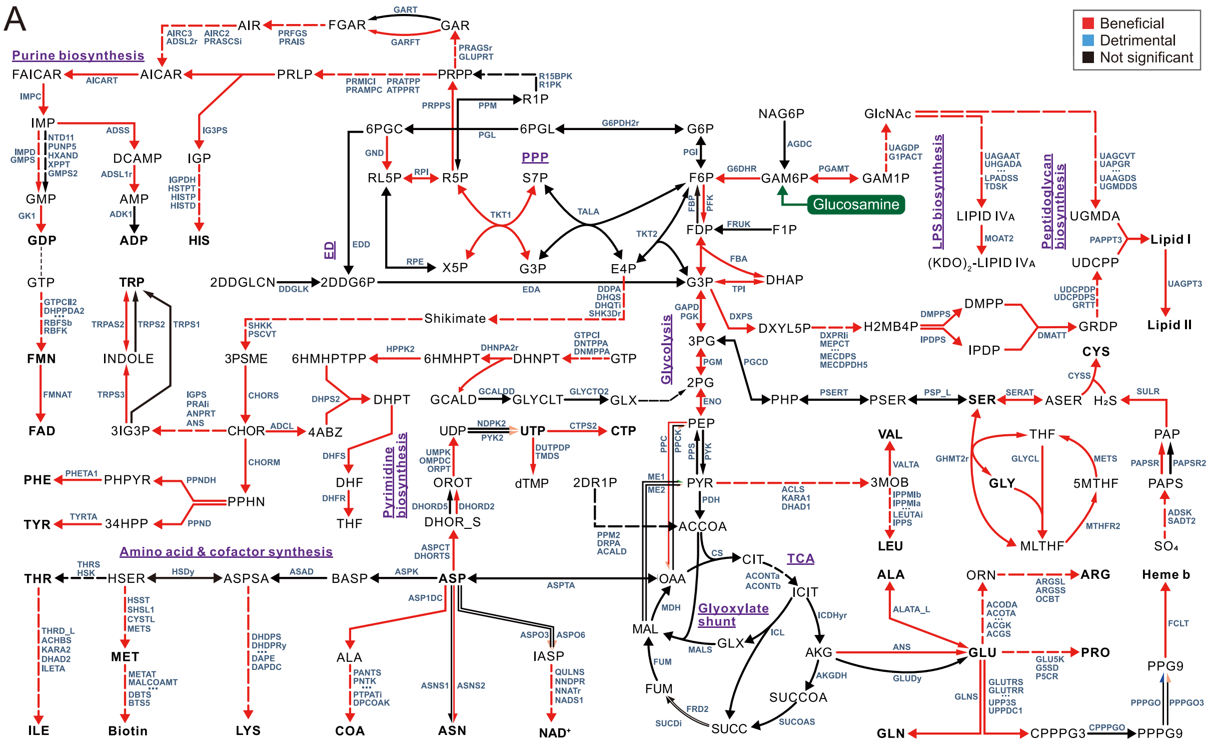

Glucose

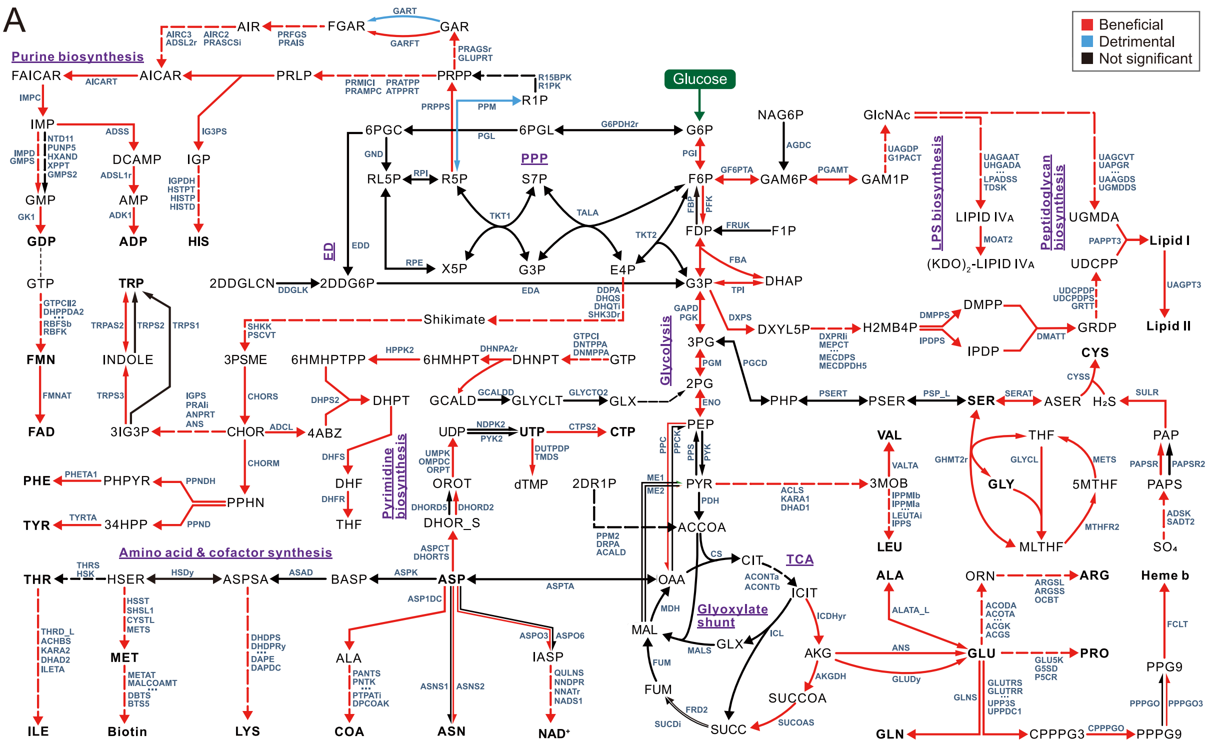

Glucuronate

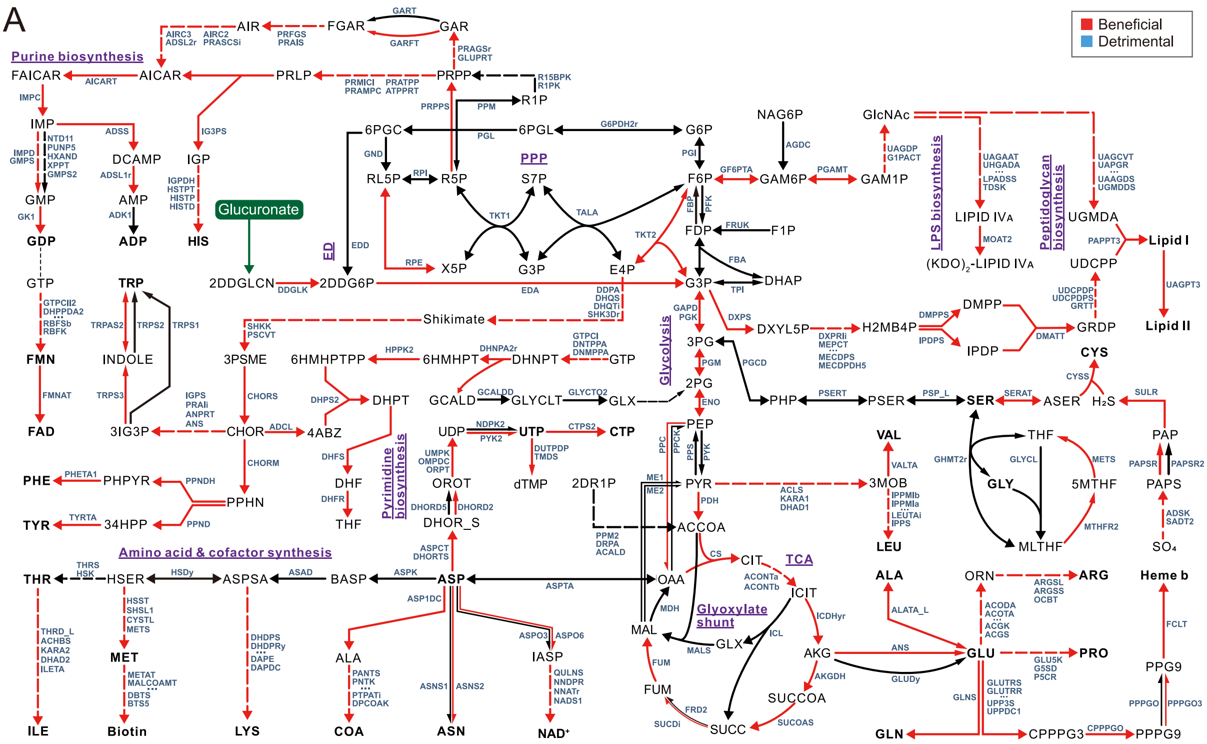
A
Beneficial
Detrimental
Glucuronate
Purine biosynthesis
FAICAR AICART AICAR PRLP PRPP R1P R15BPK R1PK
AIR AIRC3 ADSL2r AIRC2 PRASCSi FGAR PRFGS PRAIS GAR GART PRAGSr GLUPRT
PRMICI PRATPP PRAMPC ATPPRT PRPPS PPM
IMPC IMP ADSS DCAMP ADSL1r AMP ADK1 ADP
IMPD GMPS NTD11 PUNP5 HXAND XPPT GMPS2 GMP GK1 GDP
IG3PS IGP IGPDH HSTPT HISTP HISTD HIS
6PGC GND RL5P RPI R5P 6PGL PGL G6PDH2r G6P PGI
PPP S7P TKT1 TALA TKT2 X5P RPE G3P E4P
F6P FBP PFK FDP FRUK F1P GF6PTA GAM6P PGAMT GAM1P NAG6P AGDC GlcNAc
UAGDP G1PACT LPS biosynthesis UAGAAT UHGADA LPADSS TDSK LIPID IVA MOAT2 (KDO)2-LIPID IVA
Peptidoglycan biosynthesis UAGCVT UAPGR UAAGDS UGMDDS UGMDA PAPPT3 Lipid I UDCPP UAGPT3 Lipid II
ED EDD 2DDGLCN DDGLK 2DDG6P EDA FBA DHAP TPI
GTP GTPCII2 DHPPDA2 RBFSb RBFK FMN FMNAT FAD
TRP TRPAS2 TRPS2 TRPS1 INDOLE TRPS3 3IG3P IGPS PRAIi ANPRT ANS
SHKK PSCVT 3PSME Shikimate DDPA DHQS DHQTi SHK3Dr
6HMHPTPP HPPK2 6HMHPT DHNPA2r DHNPT GTPCI DNTPPA DNMPPA GTP
CHORS CHOR ADCL 4ABZ DHPS2 DHPT DHFS DHF DHFR THF
GCALDD GCALD GLYCLT GLYCTO2 GLX
GAPD PGK 3PG PGM PGCD 2PG ENO PEP Glycolysis
DXPS DXYL5P DXPRIi MEPCT MECDPS MECDPDH5 H2MB4P DMPPS IPDPS DMPP IPDP DMATT GRDP
CYS CYSS PHP PSERT PSER PSP_L SER SERAT ASER H2S SULR
CHORM PHE PHETA1 PHPYR PPNDH PPHN TYR TYRTA 34HPP PPND
Pyrimidine biosynthesis UDP NDPK2 PYK2 UTP CTPS2 CTP UMPK OMPDC ORPT DUTPDP TMDS dTMP
OROT DHORD5 DHORD2 DHOR_S 2DR1P PPM2 DRPA ACALD
PPC PPCK PPS PYK ME1 ME2 PYR PDH ACCOA ACLS KARA1 DHAD1 3MOB VALTA VAL IPPMIb IPPMIa LEUTAi IPPS LEU
GHMT2r THF GLYCL METS GLY 5MTHF MTHFR2 MLTHF
PAP PAPSR PAPSR2 PAPS ADSK SADT2 SO4
Amino acid & cofactor synthesis
THR THRS HSK HSER HSDy ASPSA ASAD BASP ASPK ASP ASPTA OAA ASPCT DHORTS
THRD_L ACHBS KARA2 DHAD2 ILETA ILE
HSST SHSL1 CYSTL METS MET METAT MALCOAMT DBTS BTS5 Biotin
DHDPS DHDPRy DAPE DAPDC LYS
ASP1DC ALA PANTS PNTK PTPATi DPCOAK COA
ASNS1 ASNS2 ASN ASPO3 ASPO6 IASP QULNS NNDPR NNATr NADS1 NAD+
CS CIT TCA ACONTa ACONTb ICIT ICDHyr MDH MAL Glyoxylate shunt ICL MALS GLX
FUM FUM FRD2 SUCDi SUCC SUCOAS SUCCOA AKGDH AKG
ALA ALATA_L ANS GLU GLUDy ORN ACODA ACOTA ACGK ACGS ARGSL ARGSS OCBT ARG
GLU5K G5SD P5CR PRO GLNS GLN GLUTRS GLUTRR UPP3S UPPDC1 CPPPG3 CPPPGO PPPG9 PPPGO PPPGO3 PPG9 FCLT Heme b

Gluconate

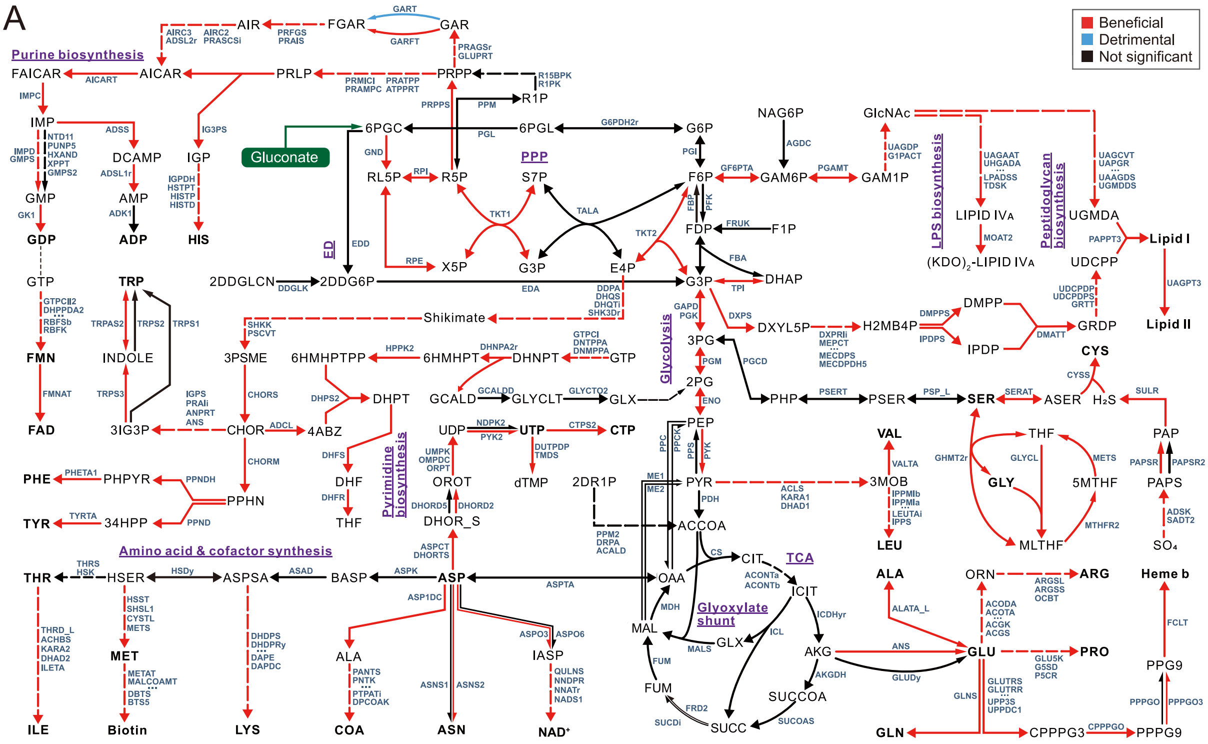

Glycerol

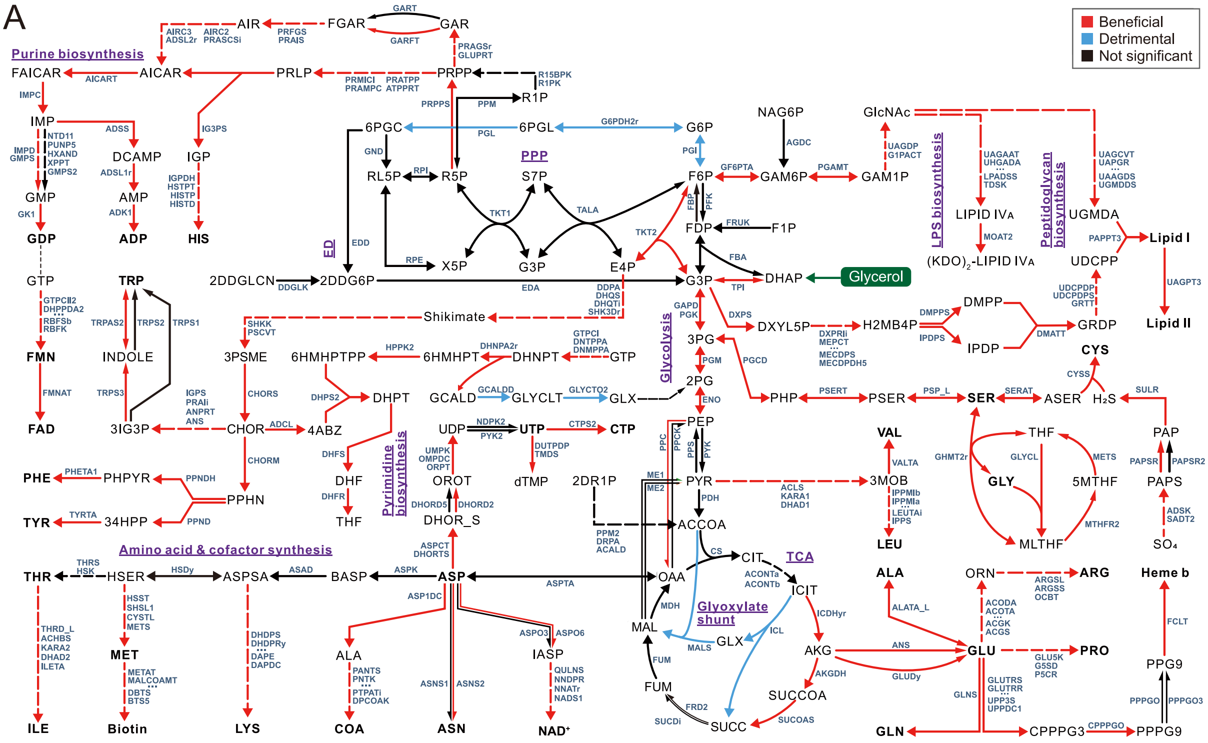

L-Alanine

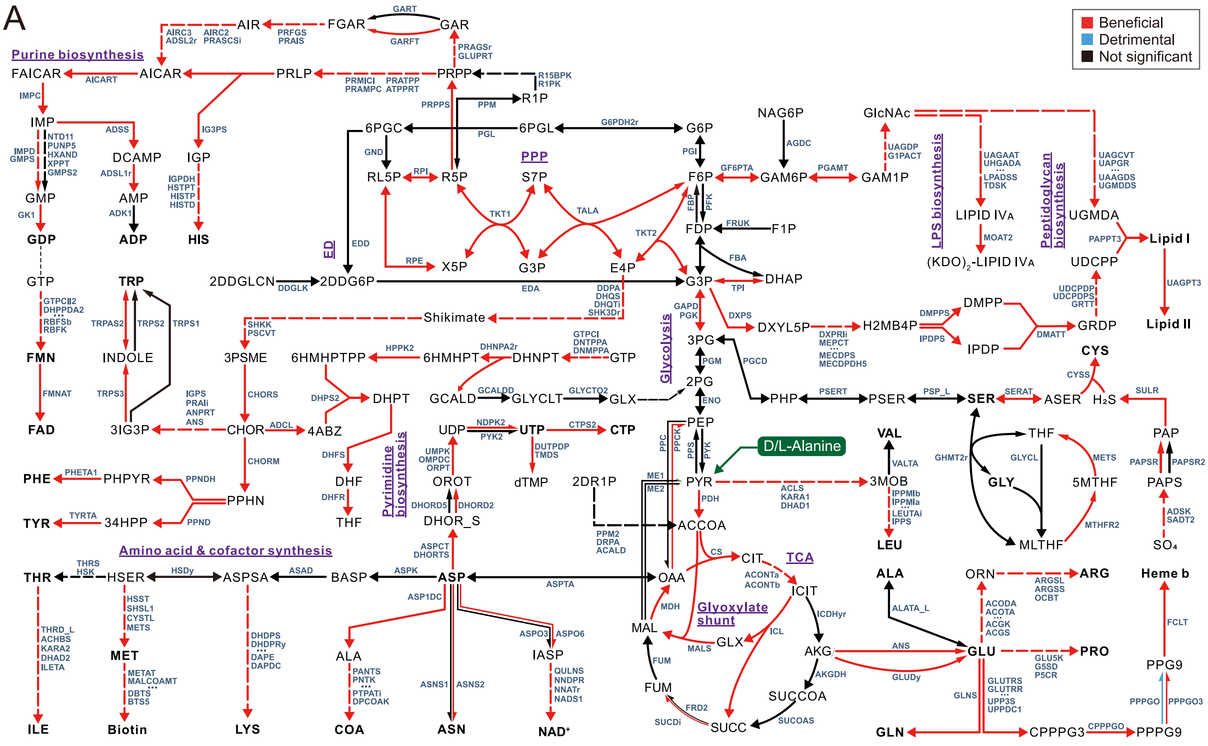

Lactate

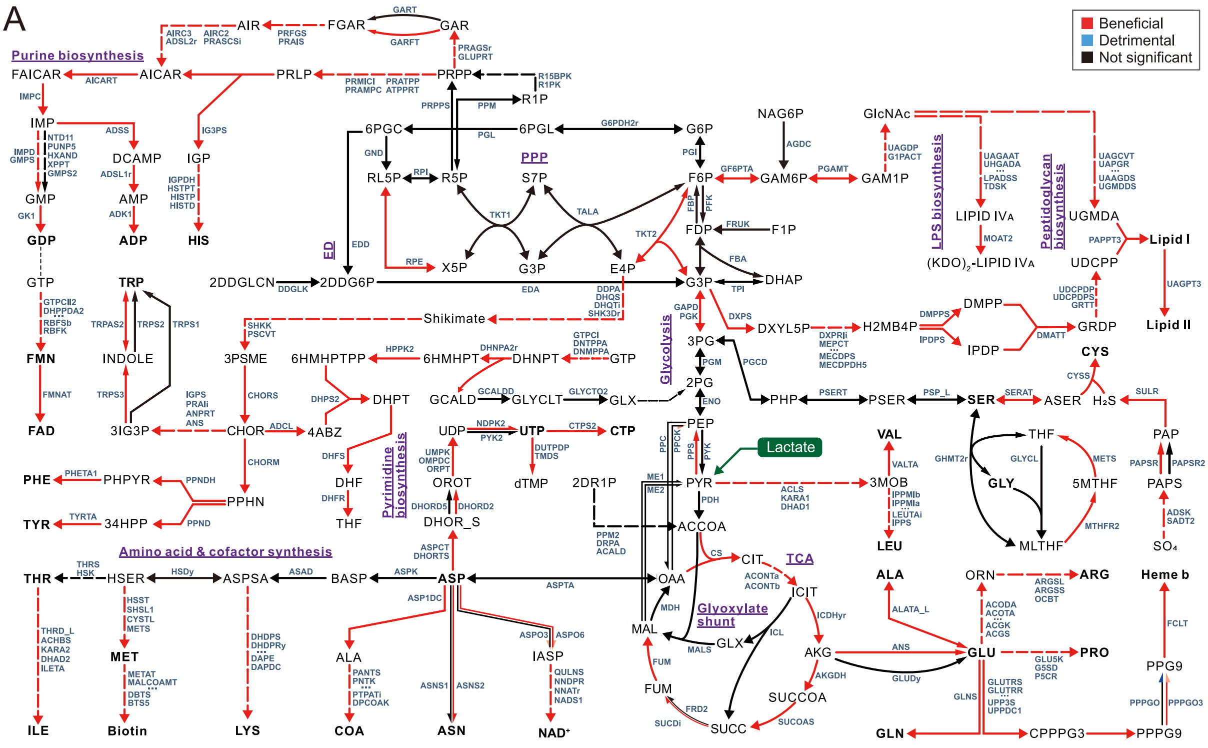

Malate

A

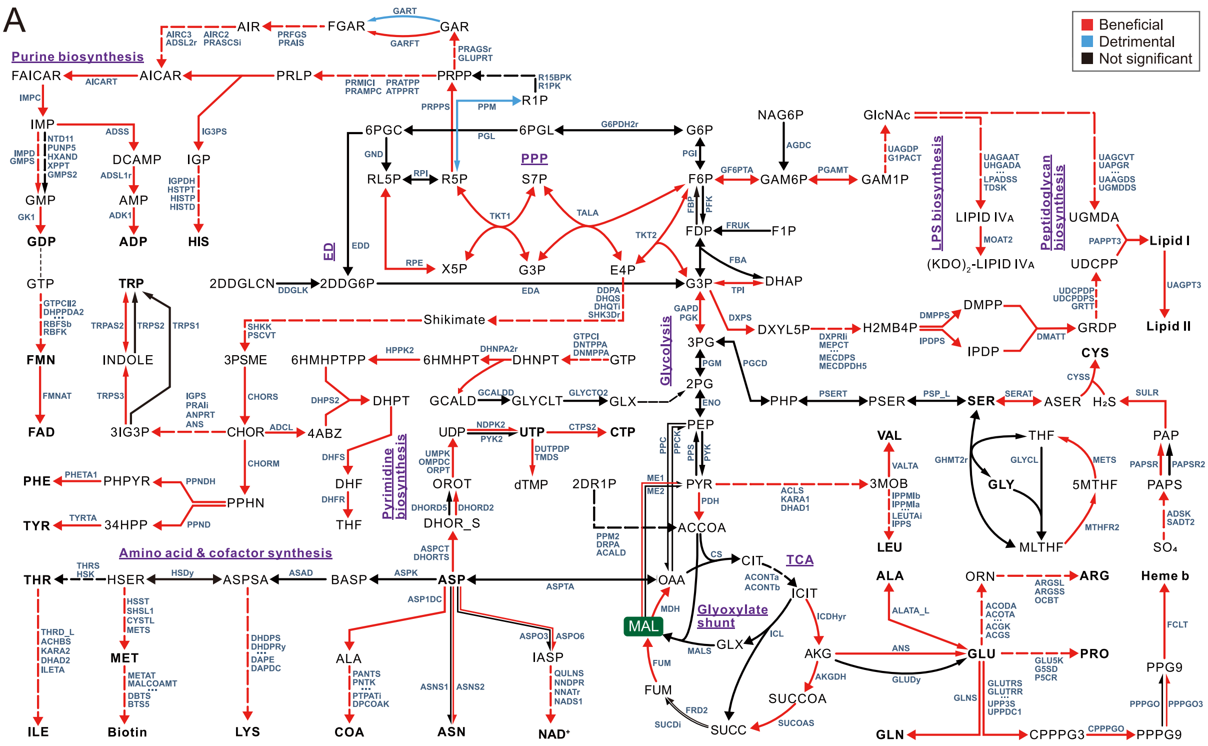

Maltose

A

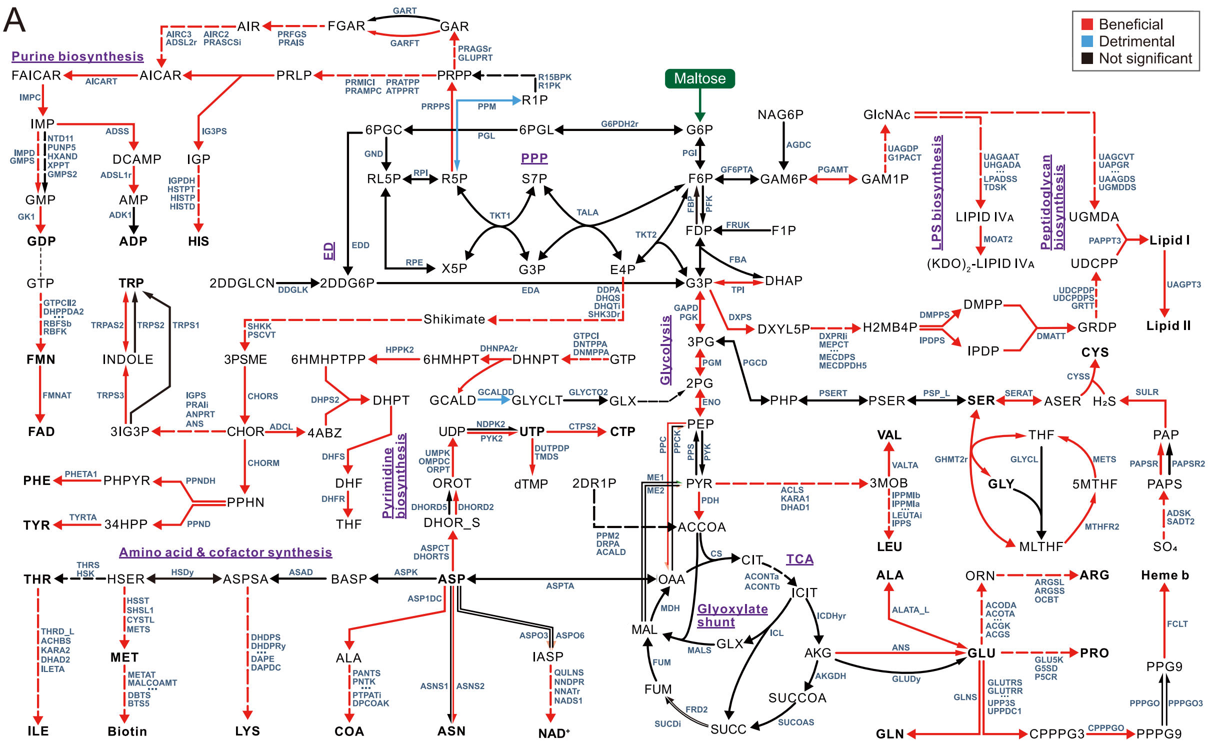

Mannitol

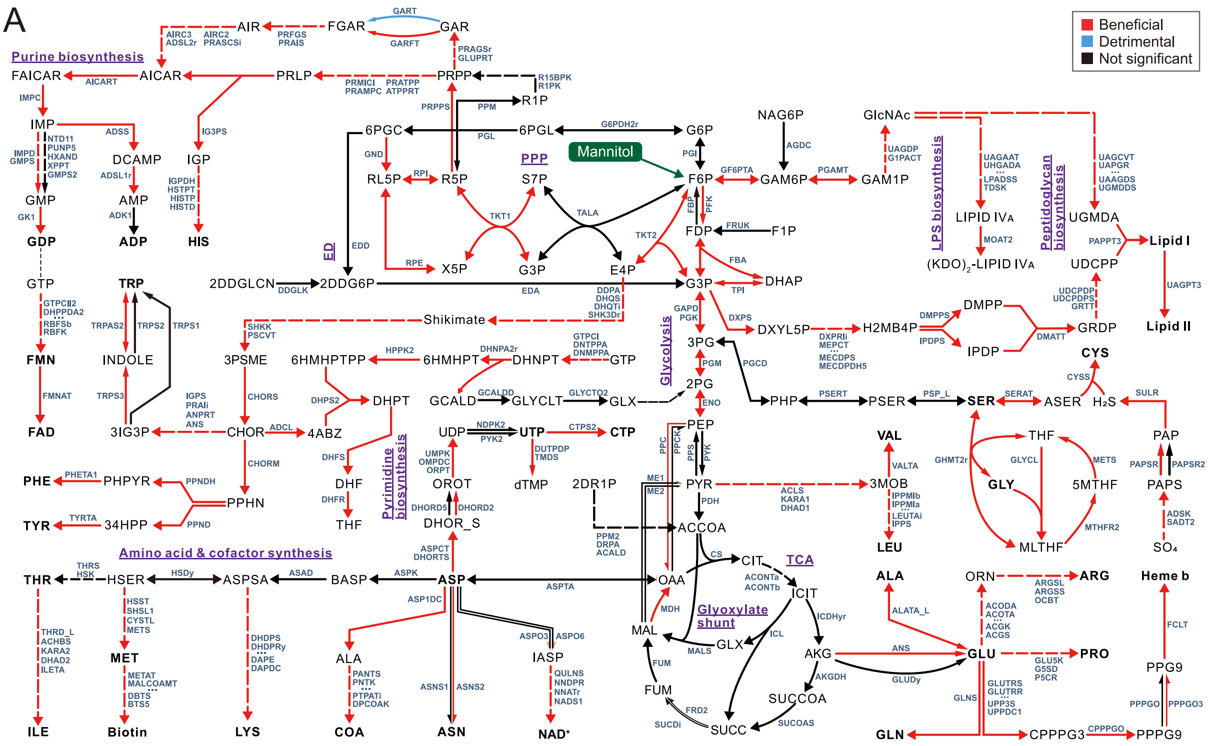

Mannose

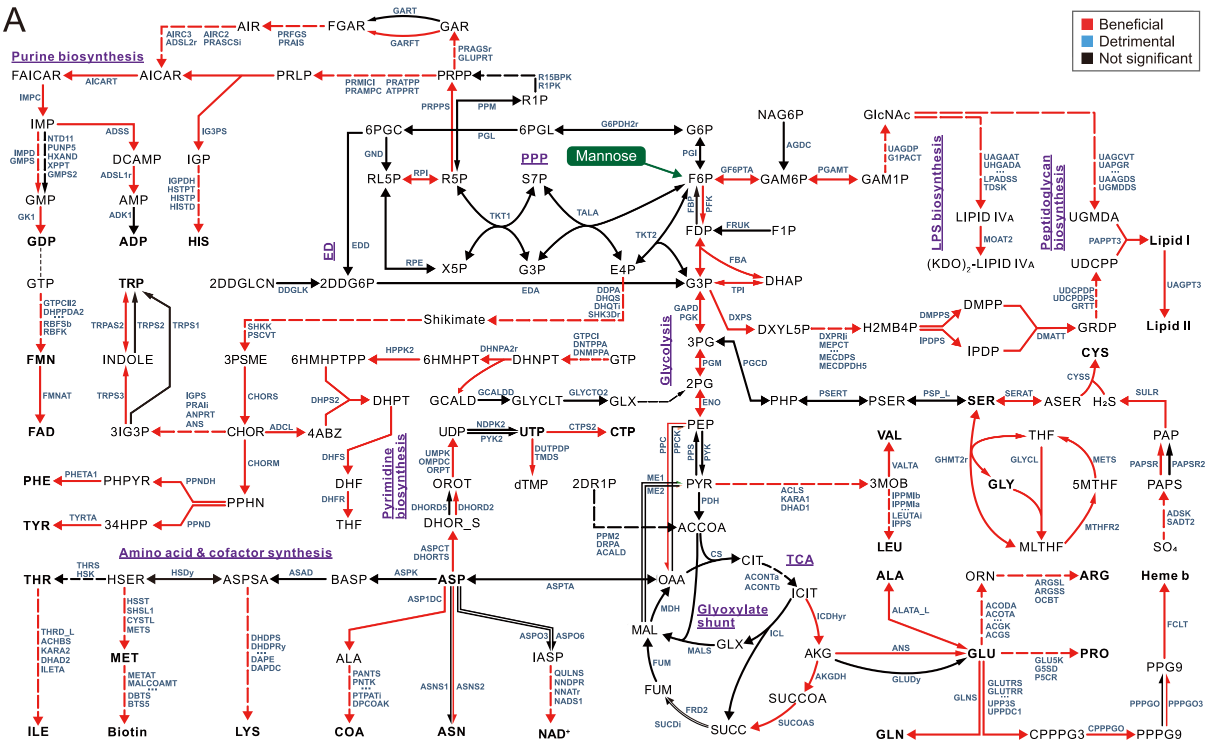

N-Acetyl glucosamine

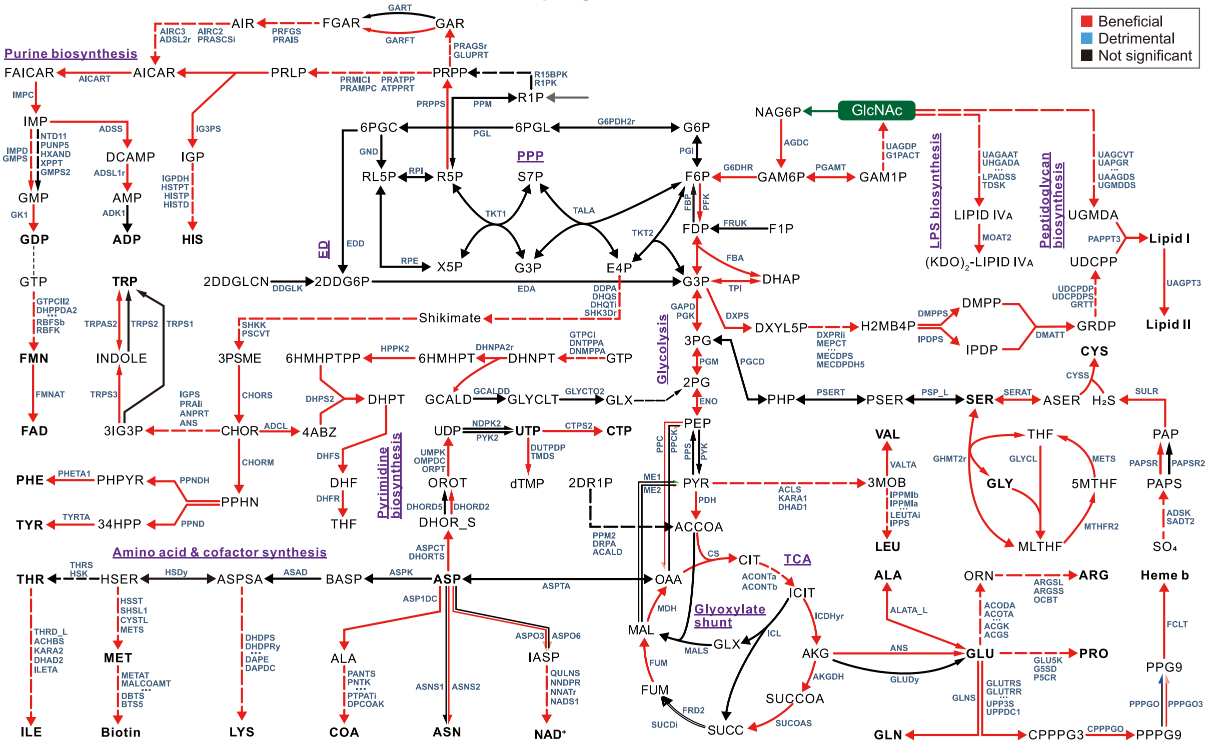

Oxaloacetate

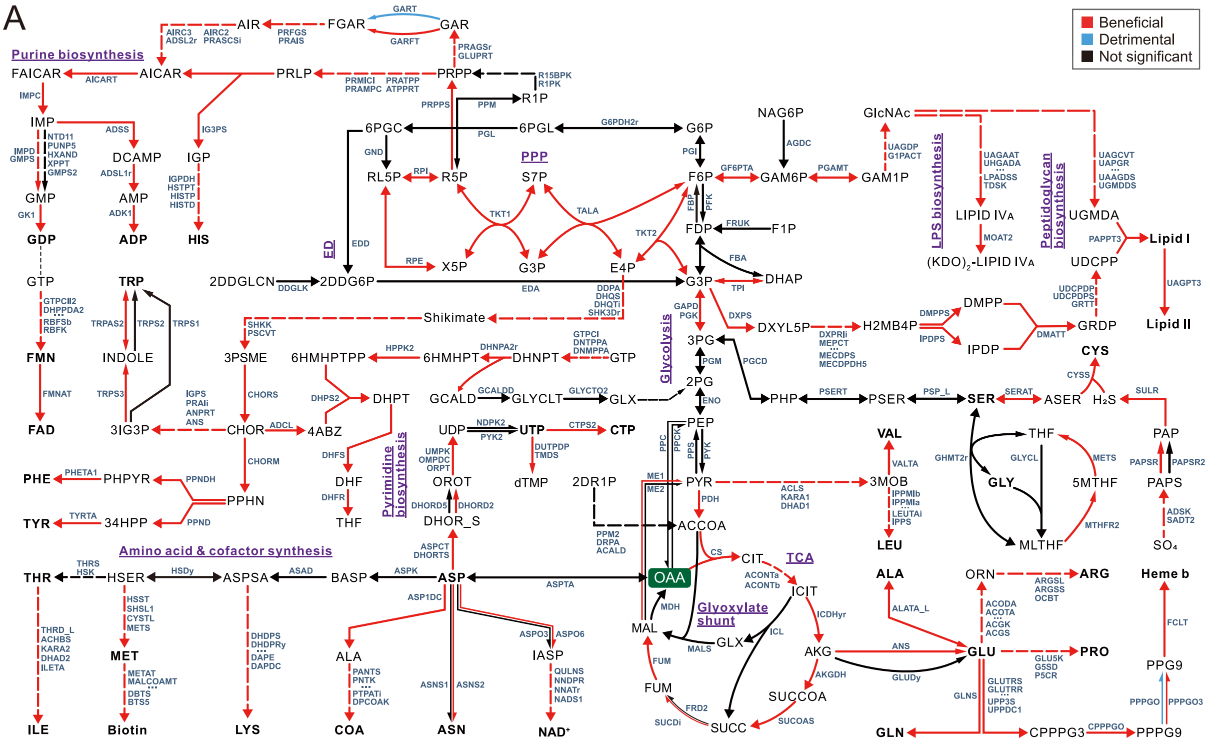

Pyruvate

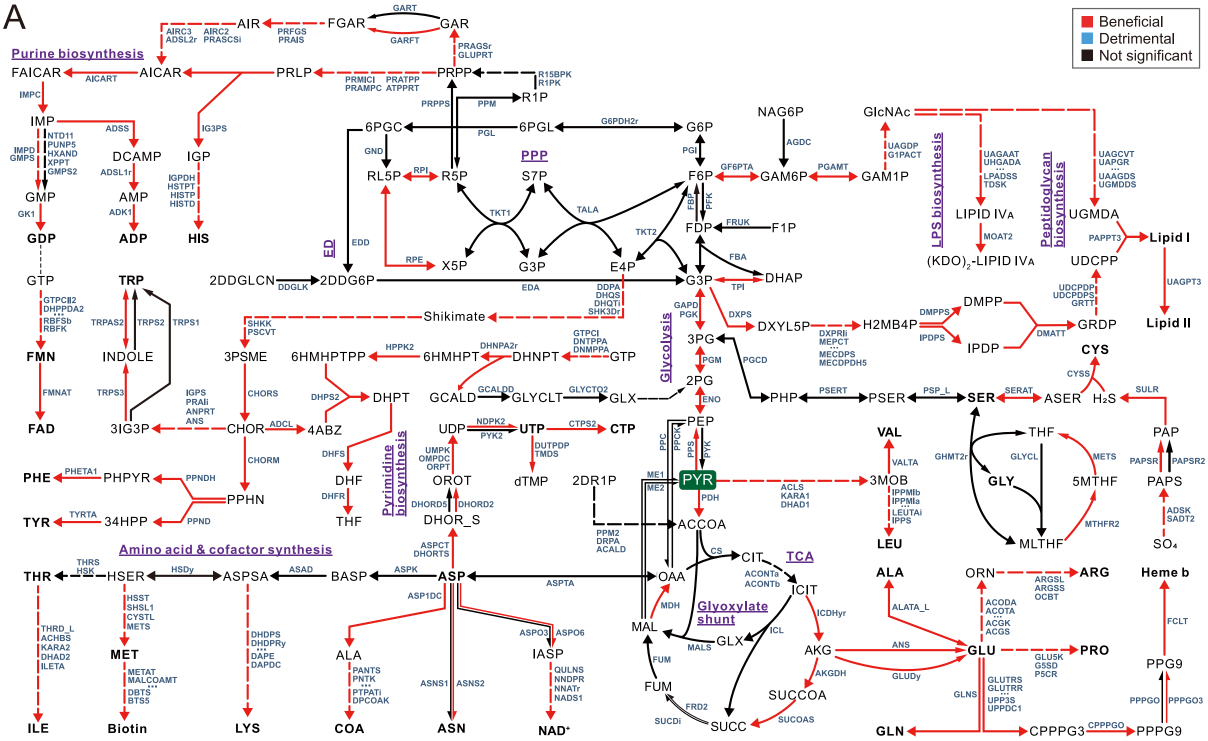

Ribose

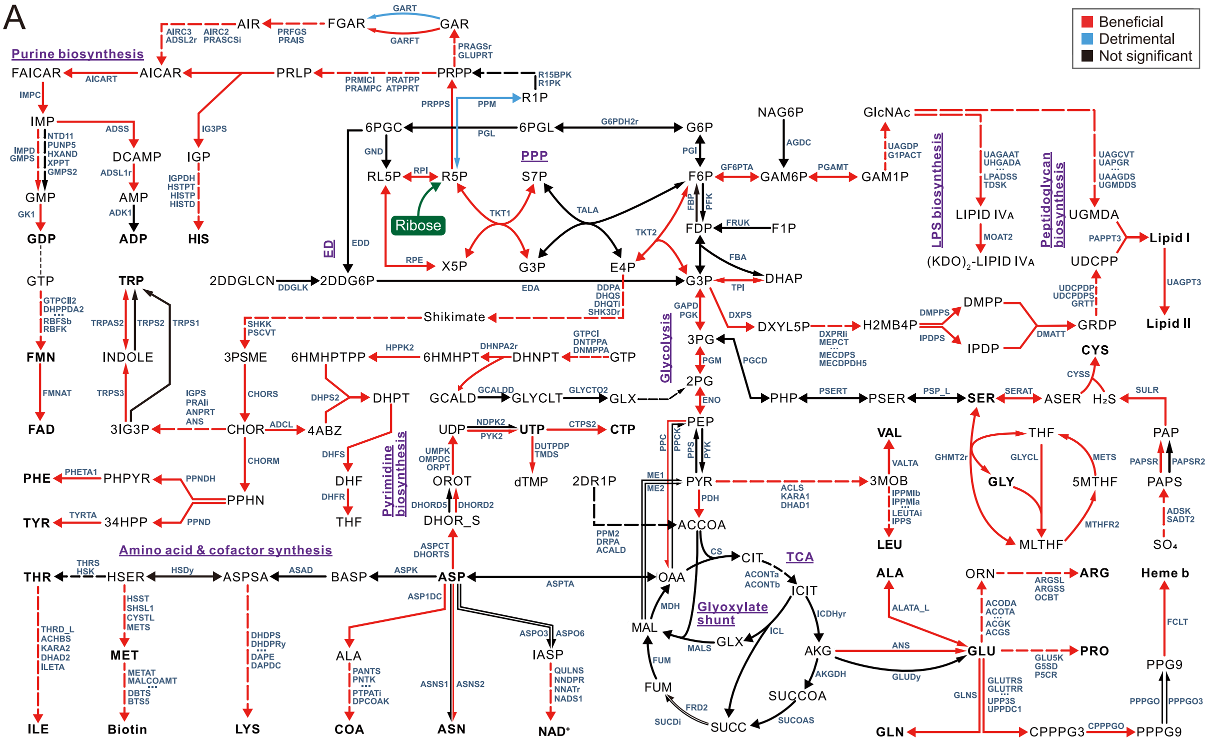

Saccharate

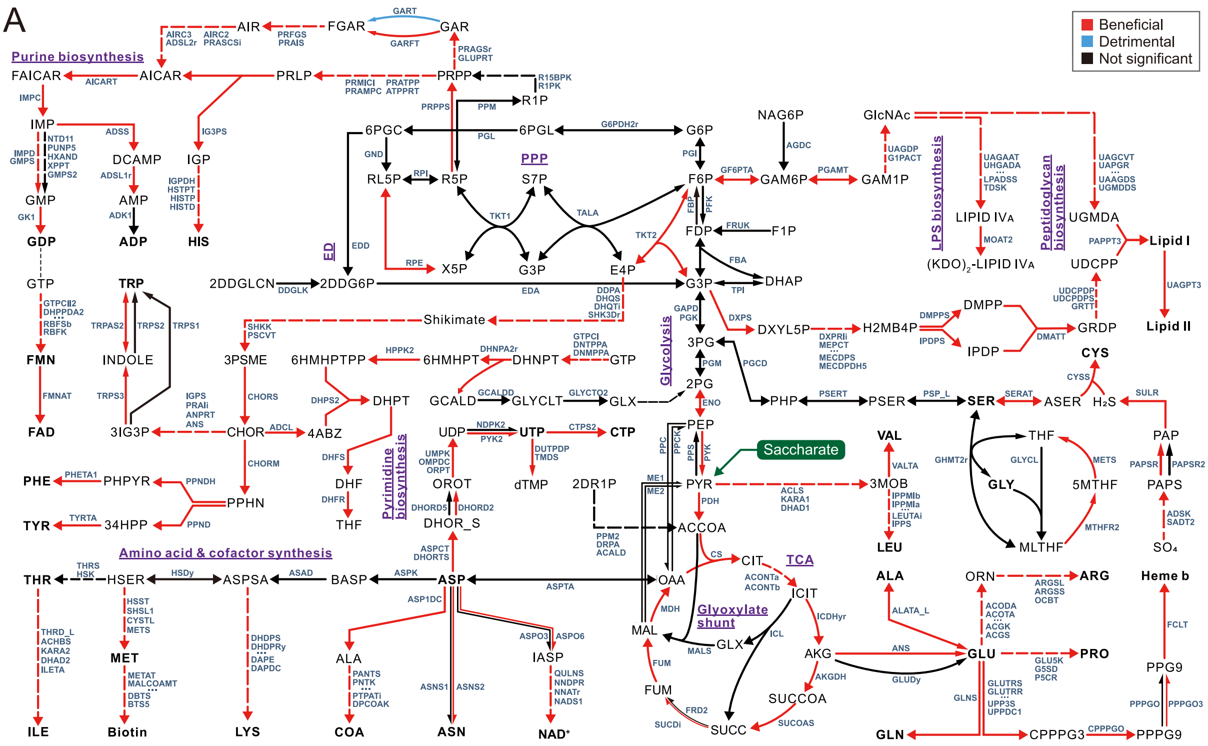

Sorbitol

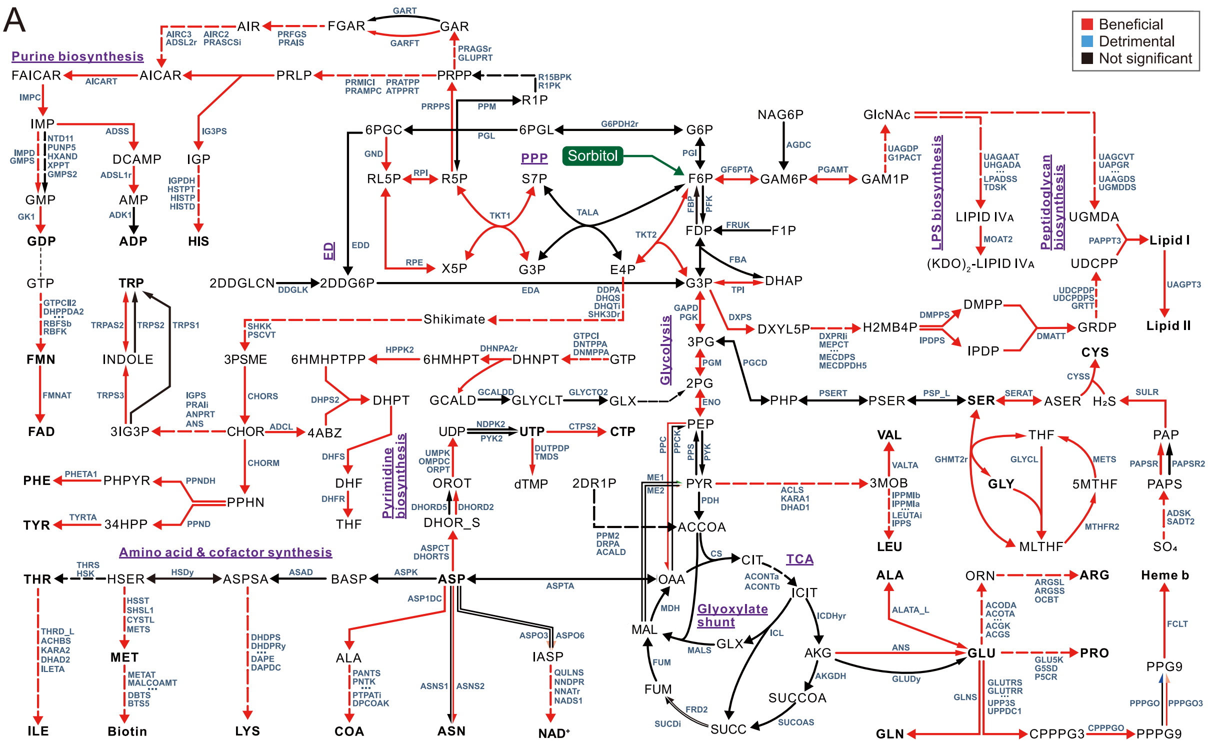

Succinate

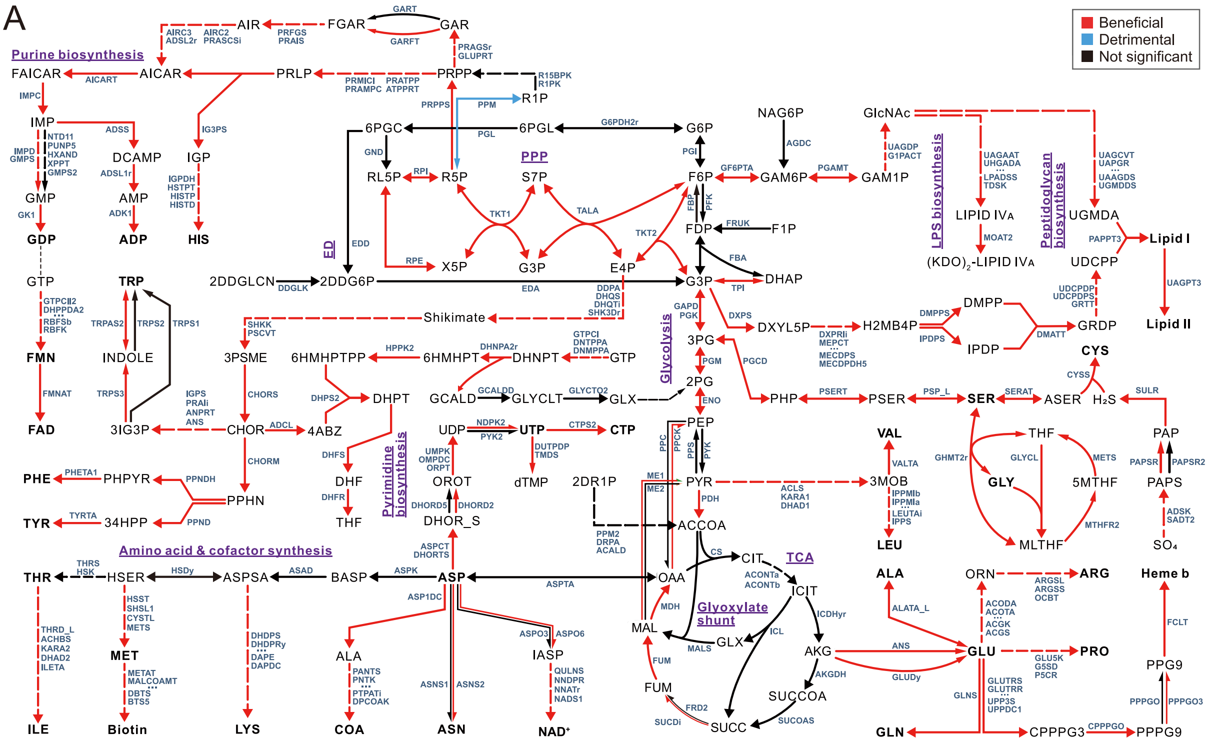

Thymidine

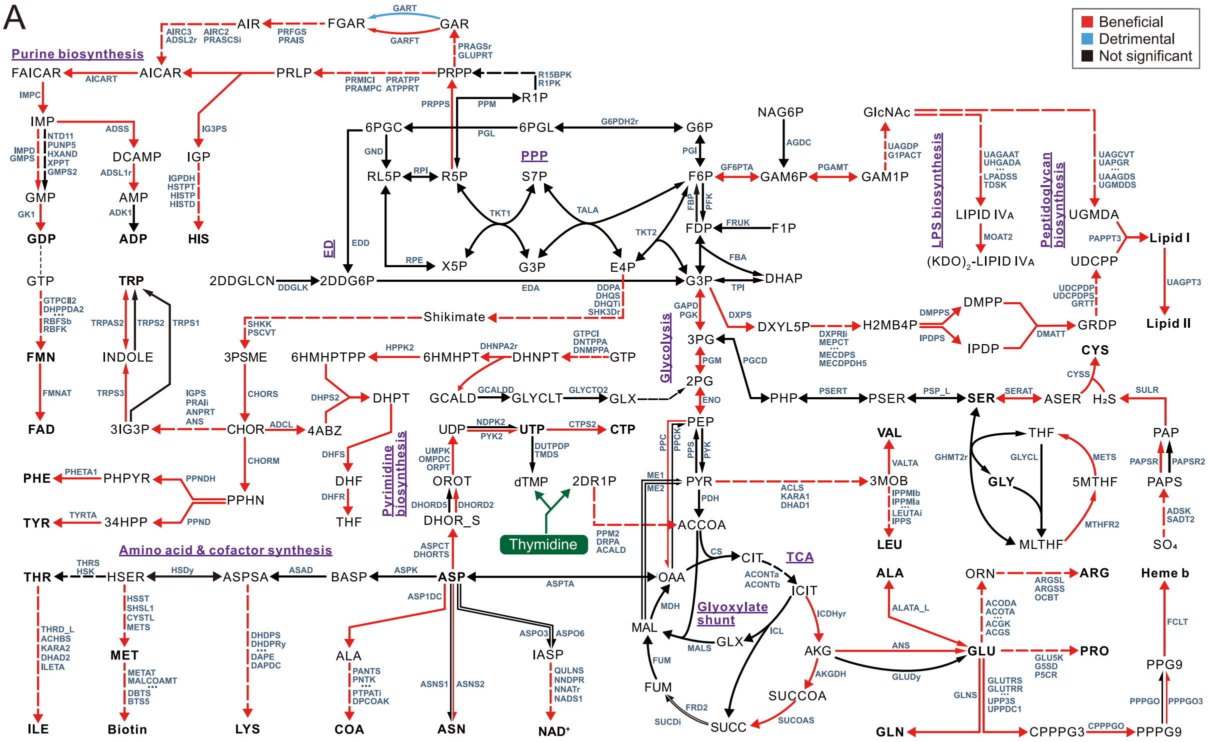

Trehalose

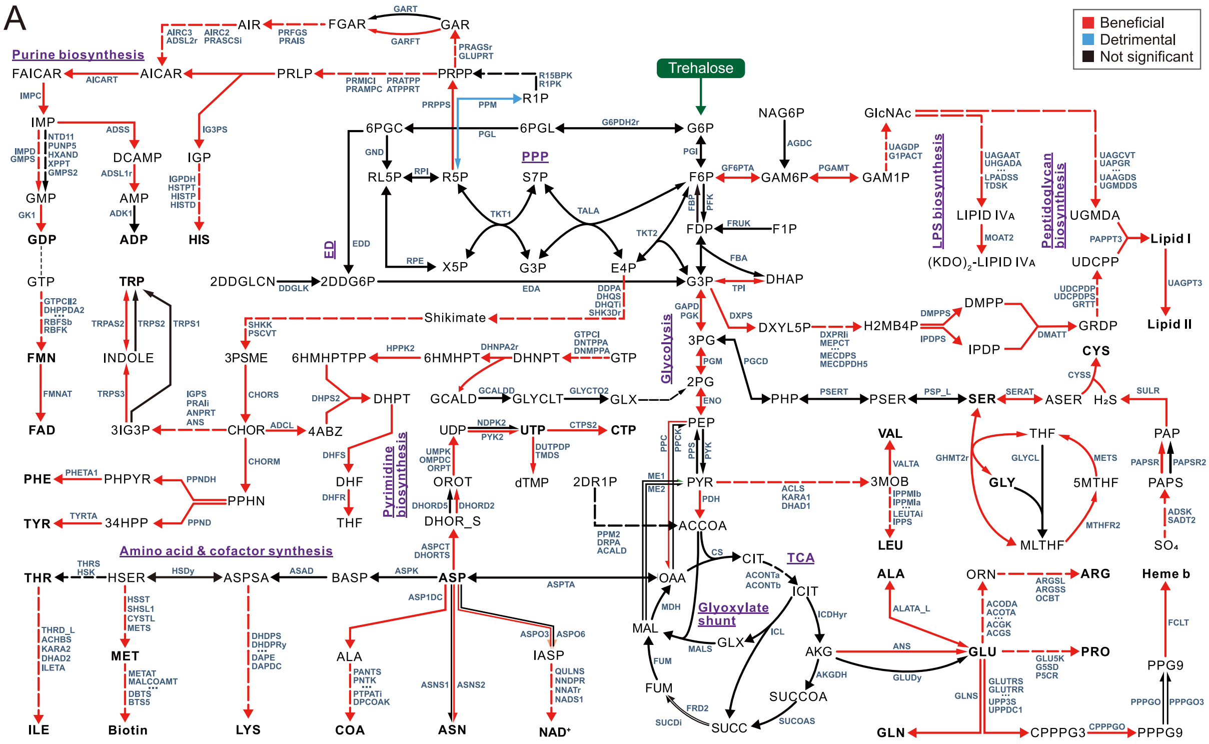

Xylose

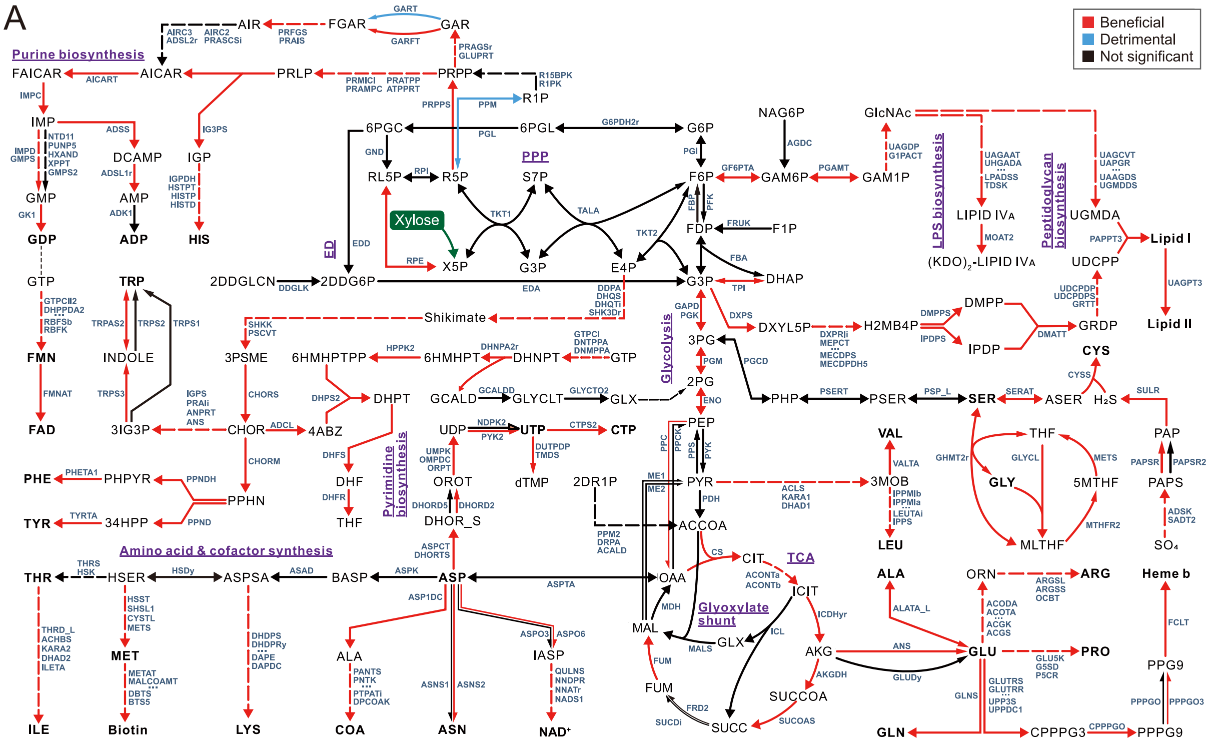

Appendix Figure S3. Growth experiment of Δ*aceB*Δ*glcB* using oleic acid as the sole carbon source.

Using MOPS minimal media supplemented with 5mM oleic acid in the presence of 0.4% Triton X-100, Δ*aceB*Δ*glcB* did not grow, in contrast to the cell growth of its parent wild-type *E. coli* K-12 BW25113. Error bars represent the standard error of the mean (SEM) from three independent cultivations.

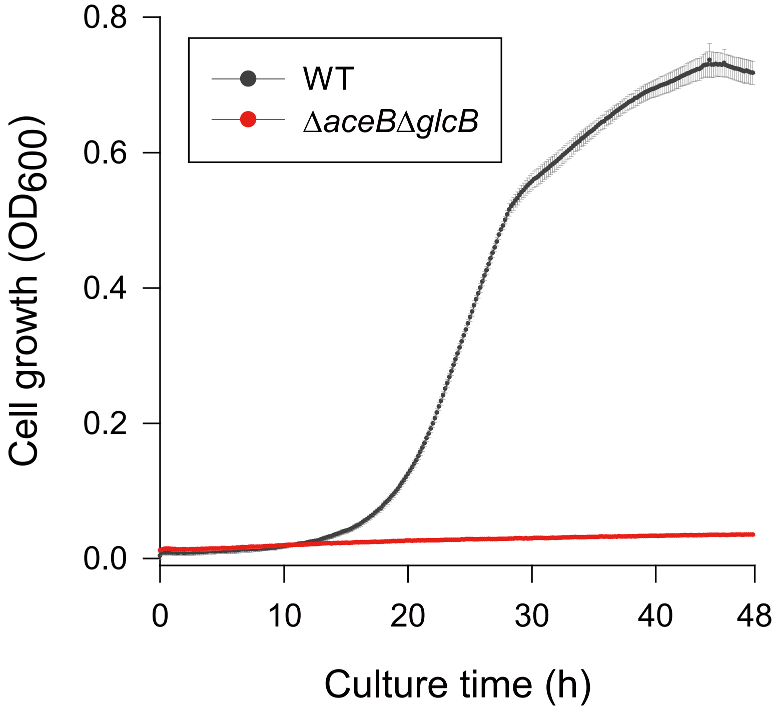

Supplement: Supplementary file 1 — Appendix [file 44320_2024_17_MOESM1_ESM.pdf]
