# Supplementary material for: Machine learning identifies key metabolic reactions in bacterial growth on different carbon sources
Source: Mol Syst Biol. 2024 Jan 30;20(3):170–86. doi: 10.1038/s44320-024-00017-w (PMC10912204; doi:10.1038/s44320-024-00017-w)
Supplement: Supplementary file 5 — Expanded View Figures [file 44320_2024_17_MOESM5_ESM.pdf]

Expanded View Figures

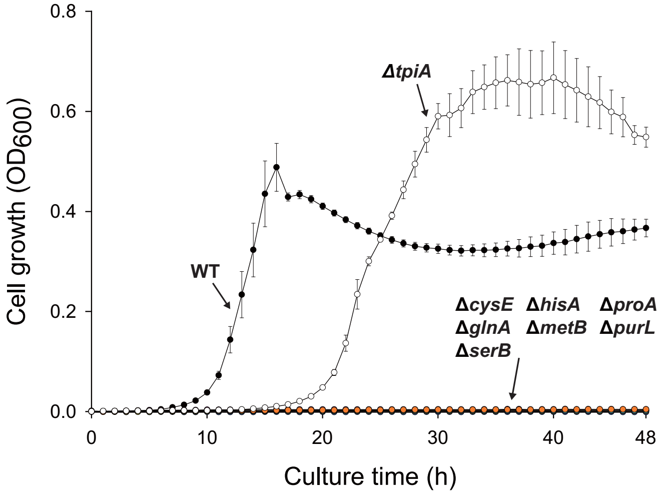

Figure EV1. Experimental validation of predicted beneficial reactions with missing deletions in the training data (Xylose condition).

The *y* axis denotes relative biomasses of gene-deletion mutants grown in the MOPS minimal medium supplemented with xylose as the sole carbon source for 48 h. Growth measurements are represented as the mean ± SEM from three independent cultivations.

© The Author(s)

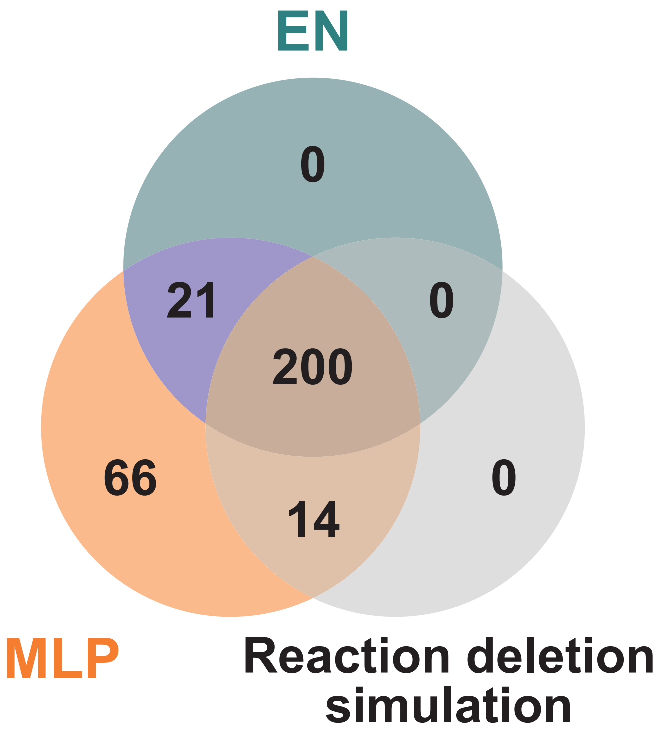

Figure EV2. Comparison of the predictions from the EN model, MLP model, and the single-reaction deletion simulation.

Metabolic reactions that were predicted to be beneficial for all 30 carbon conditions by the EN and MLP models were compared with essential reactions predicted from the simulation.

 © The Author(s)

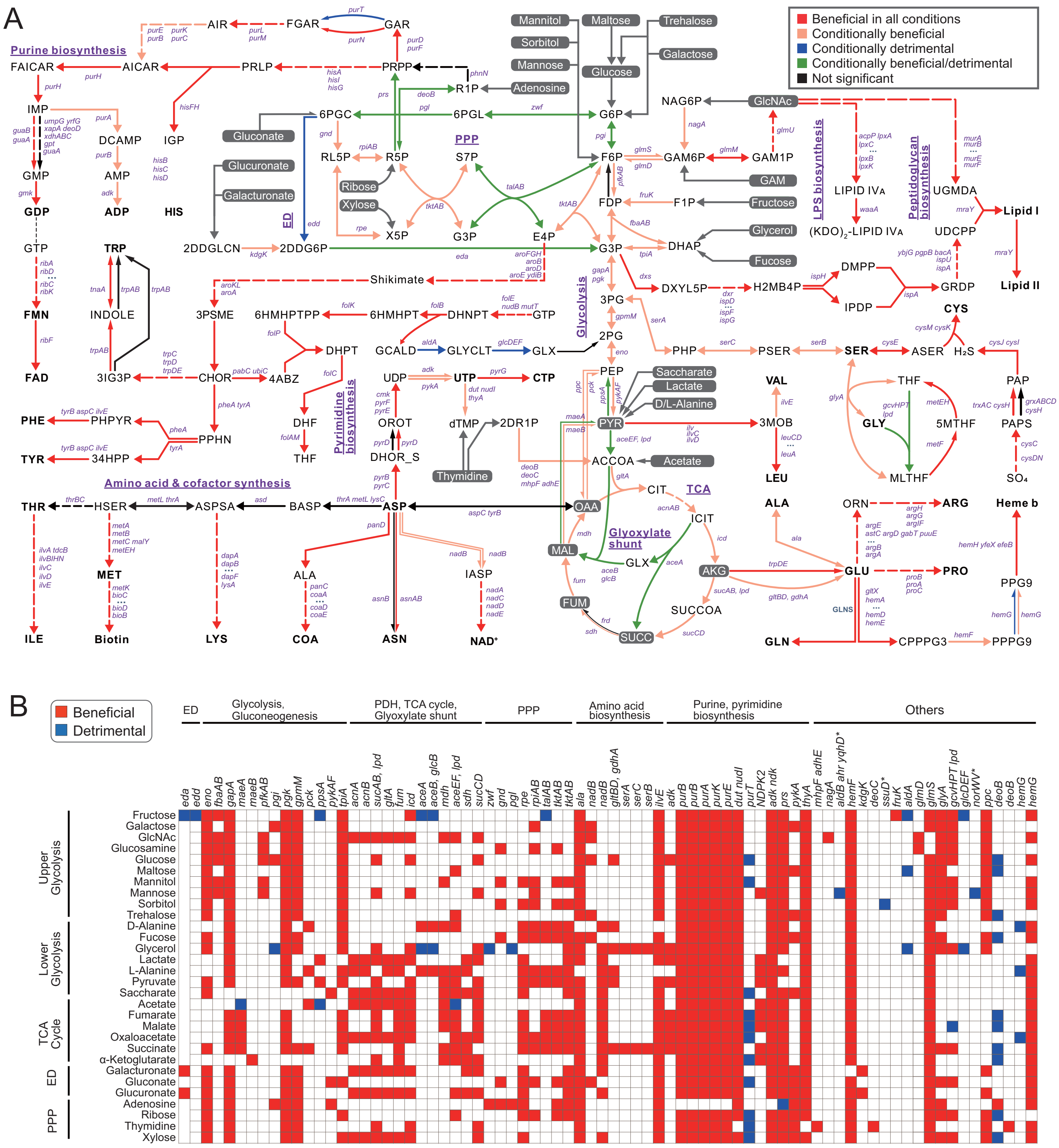

Figure EV3. Effect of the predicted metabolic genes on cell growth.

This figure is identical to Fig. 4 except that the gene names are shown instead of the reaction names. (**A**) Mapping of the model predictions onto metabolic pathways. The solid arrows indicate single metabolic reactions, and dashed arrows indicate multiple sequential reaction steps. Carbon sources are shown with a grey background. The color scheme of each arrow indicates the impact of each metabolic reaction on cell growth: red, beneficial under all 30 carbon source conditions; orange, beneficial under certain carbon conditions; blue, detrimental under certain carbon conditions; green, beneficial or detrimental under certain carbon conditions; black, not significant. (**B**) Heatmap representation of the impact of the model predictions on cell growth according to the carbon source. Within each cell, the predicted beneficial and detrimental reactions are colored red and blue, respectively. Metabolic genes not shown in panel A are marked with an asterisk. For clarity, genes that were predicted to be beneficial across all carbon source conditions are not shown.
